# Therapeutic effect and safety of curcumin in women with PCOS: A systematic review and meta-analysis

**DOI:** 10.3389/fendo.2022.1051111

**Published:** 2022-10-27

**Authors:** Wenjuan Shen, Yangfan Qu, Huan Jiang, Hongwei Wang, Yujia Pan, Yuehui Zhang, Xiaoke Wu, Yanhua Han, Yang Zhang

**Affiliations:** ^1^ Department of Obstetrics and Gynecology, First Affiliated Hospital of Heilongjiang University of Chinese Medicine, Harbin, China; ^2^ Department of Obstetrics and Gynecology, Heilongjiang University of Chinese Medicine, Harbin, China; ^3^ Department of Traditional Chinese Medicine, Cixi People’s Hospital Medical and Health Group, Cixi, China; ^4^ Department of Internal Medicine, First Affiliated Hospital of Heilongjiang University of Chinese Medicine, Harbin, China

**Keywords:** curcumin, polycystic ovary syndrome, meta-analysis, systematic review, complementary therapy

## Abstract

**Background:**

Polycystic ovary syndrome (PCOS) is a multi-factorial heterogeneous syndrome that has both adverse reproductive and metabolic implications for affected women and its management is a challenging clinical problem. Curcumin, as a phenolic compound with potent anti-inflammatory and antioxidant properties exerting positive effects on the lipid profile and insulin resistance, appears to be a valuable treatment regimen for patients with PCOS.

**Objective:**

This study aimed to evaluate the efficacy and safety of curcumin in the treatment of PCOS.

**Methods:**

Chinese databases (Chinese National Knowledge Infrastructure, China Biology Medicine Databases, VIP database, Wanfang Database, and Chinese Clinical Trial Registry) and English databases (PubMed, Web of Science, Embase, Cochrane Library, Scopus and Clinical trials) were thoroughly investigated through screening randomized controlled trials on curcumin in PCOS published from the date of inception to May 2022. Standardized data search and abstraction were conducted following the preferred reporting items for systematic reviews and meta-analysis (PRISMA) statement. Quantitative and qualitative analyses were performed. Heterogeneity was assessed using I^2^ statistics.

**Results:**

A total of 447 patients from seven randomized controlled trials were included in the meta‐analysis. Results showed that the ingestion of curcumin decreased body mass index (WMD -0.267, 95% CI -0.450 to -0.084, P = 0.004, I^2^ = 0.0%), fasting plasma glucose (WMD -3.618, 95% CI -5.165 to -2.071, P < 0.001, I^2^ = 20.4%), insulin (WMD -1.834, 95% CI -2.701 to -0.968, P < 0.001, I^2^ = 8.4%), homeostatic model assessment for insulin resistance (WMD -0.565, 95% CI -0.779 to -0.351, P < 0.001, I^2^ = 0.0%), total cholesterol (WMD -15.591, 95% CI -27.908 to -3.273, P = 0.013, I^2^ = 68.9%), C-reactive protein (WMD -0.785, 95% CI -1.553 to -0.017, P = 0.045, I^2^ = 23.9%), and increased the quantitative insulin sensitivity check index (WMD 0.011, 95% CI 0.005 to 0.017, P = 0.001, I^2^ = 39.6%). As for safety, the treatment group did not cause significant adverse reactions than that in the control group.

**Conclusion:**

In light of presented findings, curcumin has beneficial effects on serum markers of inflammation, weight loss and glucose and lipid metabolism in patients with PCOS. The incidence of adverse reactions does not increase with the application of curcumin. However, a larger, more definitive study is needed to further investigate these results.

**Systematic review registration:**

https://www.crd.york.ac.uk/prospero/, identifier CRD42022332394.

## Background

Polycystic ovary syndrome (PCOS), the most common endocrine disorder, is characterized by ovulatory dysfunction, hyperandrogenism, and polycystic ovaries. At present, the incidence of PCOS is from 6% to 25% in women of reproductive age worldwide (1, 2), of which the prevalence in China is 7.8%, and it has increased by 65% in the past 10 years (3). In addition, PCOS has been linked to a number of higher risks of metabolic disorders, including insulin resistance (IR), glucose intolerance, type 2 diabetes, obesity, dyslipidemia, and cardiovascular diseases (4). Among them, cardio metabolic diseases such as myocardial infarction and stroke, are major causes of death in women (5).

As a global epidemic disease, obesity is a 21st-century major public health challenge (6). Despite adiposity is not a defining criterion for PCOS, rates of obesity are estimated to be 2.8 times higher in PCOS than in the general population, with a prevalence of 50-80% (7). Excess adiposity, particularly around the abdomen, causes insulin resistance, a critical etiological component to PCOS (8, 9). Insulin resistance and consequent hyperinsulinemia lead to hyperandrogenism by acting on the adrenal gland, ovaries and liver to increase androgen production and decrease sex hormone binding globulin (SHBG) (10). In addition, androgen excess has been shown to induce visceral fat accumulation and possibly adipose tissue dysfunction (11). Meanwhile, some studies have found hyperandrogenism aggravates the symptoms of insulin resistance, leading to a vicious cycle that promotes PCOS development.

Over the recent years, numerous preclinical and clinical studies have demonstrated that PCOS is associated with a chronic inflammatory state, inflammatory cytokines in PCOS patients can induce adipocyte proliferation by modulation of signal transducer and activator of transcription 3 (STAT3) signaling (12, 13). Excessive inflammatory factors also produce redundant reactive oxygen species (ROS) and disrupt internal ROS homeostasis, thereafter inhibit insulin signaling and insulin-mediated glucose transport, aggravating insulin resistance (14). Decreasing plasma insulin level and ameliorating insulin resistance not only leads to an improvement in reproductive abnormalities, but also probably reduces the future risk of developing diabetes and cardiovascular disease in PCOS women (15). Besides lifestyle intervention, metformin, a biguanide, is a commonly prescribed agent for the management of PCOS (16). It works by inhibiting hepatic glucose production, reducing intestinal glucose absorption and improving glucose metabolism (17). However, it has been observed that 20-30% of people receiving metformin therapy develop gastrointestinal side effects, with approximately 5% being unable to tolerate metformin at all (18). Fortunately, complementary and phytomedicines medicines have shown satisfactory results to cure PCOS.

Curcumin (diferuloylmethane) is a natural polyphenol extracted from the roots of Curcuma longa (Zingiberaceae). For many years, as an Indian spice, it has been widely used as food additives, food pigments and seasonings (19). In view of its anti-inflammatory, hypolipidemic and anti-anxiety activities, it is also used to treat a variety of chronic diseases, such as diabetes, depression and so on (20). With the deepening of research, a lot of evidence shows that curcumin is a natural regulator and protector in the process of female reproduction (21). Continuous (up to 4 months) and high-dose (up to 12 grams in human body) use of curcumin is also quite safe (22, 23). Curcumin has obvious protective effect on ovarian tissue. In fact, this compound seems to be involved in inhibiting the expression of vascular endothelial growth factor (VEGF), a proangiogenic factor closely related to the formation of PCOS, thereby inhibiting ovarian angiogenesis, preventing ovarian fibrosis and promoting matrix degradation (24). Nanocurcumin can significantly improve oxidative markers, glucose index and tumor necrosis factor α (TNF-α) level, restore phosphoinositol 3 kinase (PI3k)/threonine kinase (Akt)/mammalian target of rapamycin (mTOR) level, and then reduce insulin resistance and maintain the integrity of islet function (25).

Many clinical trials have shown that curcumin supplementation has a beneficial effect on improving insulin levels (26, 27). The latest clinical trial on the potential effectiveness of curcumin on PCOS also unanimously showed that Curcuma longa (CL) can increase insulin sensitivity in patients with PCOS (28). Although these findings are not supported by other studies (29). And most of the existing systematic reviews have observed the efficacy of curcumin and paid more attention to the effect of curcumin on blood glucose control and blood lipid level of PCOS, but there is a lack of the latest systematic review to evaluate safety of curcumin as an intervention group. Given the growing interest in alternative and complementary therapies and the global burden of PCOS, we attempt to provide an updated summary of the efficacy and safety on PCOS.

## Materials and methods

This systematic review was conducted following the Preferred Reporting Items for Systematic Review and Meta-analysis (PRISMA) statement (30) and the study protocol was registered on PROSPERO (CRD42022332394).

### Search strategy

Eligible literature published up to May 2022 was identified through a search in PubMed, Embase, Cochrane Library, Web of Science, Scopus, Clinical Trials, Chinese Clinical Trial Registry, Chinese Biomedical Literature Database (CBM), Chinese National knowledge Infrastructure (CNKI), VIP database, and Wanfang Database, and an additional search of grey literature and missed references to help minimise publication bias. The search strategy consisted of medical subject headings (MeSH) as well as free words and was slightly adjusted for the syntax appropriate for the different databases without restriction to race, ethnicity, or language. Details of the search strategies are presented in the **Supplementary Appendix 1**.

### Eligibility criteria

Studies were considered eligible if they met the following criteria: 1) parallel-assignment randomized controlled trials (RCTs) of evaluation of the effects of curcumin on PCOS; 2) all patients, at any age, had PCOS as classified by the revised European Society for Human Reproduction and Embryology/American Society for Reproductive Medicine (ESHRE/ASRM) diagnosis, which were based on the Rotterdam criteria; 3) the interventions included curcumin/Curcuma longa or curcumin/Curcuma longa combined with medication (unlimited dosage form, dose, or duration); 4) the control group should be placebo or medication; and 5) the trial gives enough information to conduct the effect estimates for meta-analysis. Exclusion criteria were as follows: 1) editorials, reviews, book chapter, letter, meta-analyses, observational study, animal experiments and so on; 2) women who had other pathologies such as congenital adrenal hyperplasia, Cushing’s syndrome, thyroid hormone abnormalities, hyperprolactinemia, ovarian/adrenal tumors or any severe medical problem or any neurological or psychiatric history.

Two investigators independently performed the eligibility assessment on the basis of inclusion, and any disagreement was resolved by discussion. After deletion of duplicates, they screened all titles and abstracts for primary screening. Subsequently, the full texts of remaining articles were scrutinized to determine eligible studies.

### Data extraction

Two researchers independently scrutinized each eligible article, extracted data and cross-checked the results to ensure the data accuracy. Any discrepancy was resolved through discussion to reach consensus. The following parameters were collected from each study: basic information of the articles (first author, publication year, country), participants (race, mean age, and sample size), curcumin characteristics (dose, frequency, treatment duration and route of administration), comparison methods, every outcome parameter and adverse effects. For studies with missing or ambiguous data, if possible, we will attempt to contact the first or corresponding author *via* telephone or email for clarification or addition to ensure the integrity of the data.

### Risk-of-bias assessment

Two authors used the Cochrane risk of bias tool to assess methodological quality of RCTs, which included the following seven specified domains: random sequence generation, allocation concealment, blinding of participants and personnel, blinding of outcome assessment, incomplete outcome data, selective reporting, and other bias. Each reviewer appraised bias according to the specific content within each item, designating a low, high, or unclear risk of bias by answering yes, no or unclear. Disagreements between the two reviewers were resolved through discussion or by consulting a third author until there was 100% agreement.

### Statistical analyses

All statistical analyses were conducted with Stata software, version 14.0 (StataCorp) in accordance to the guidelines described in the Cochrane Handbook for systematic reviews of interventions. For dichotomous variables, the odds ratio (OR) with corresponding 95% confidence intervals (CIs) was calculated to summarize the difference between the groups. For continuous data, the results were presented as weighted mean difference (WMD) together with 95% CI of changes before and after the therapy in the curcumin group with those in the control group. Since some studies used different measures for the same outcome (e.g., AST and ALT), we calculated the standardized mean difference (SMD) (31). Heterogeneity among the included studies was estimated using Q statistic and the I2 statistic, results were deemed as low heterogeneity (I2 < 25%), medium heterogeneity (I2 = 25%-50%), or high heterogeneity (I2 > 50%) (32). Owing to the clinical heterogeneity inherent in our data such as ethnic differences, different use of curcumin preparations as well as duration of treatment, and so forth, random-effects models were performed for calculating pooled effect measures. We also conducted a sensitivity analysis to test the robustness of the findings.

## Results

### Study selection

**Figure 1:**
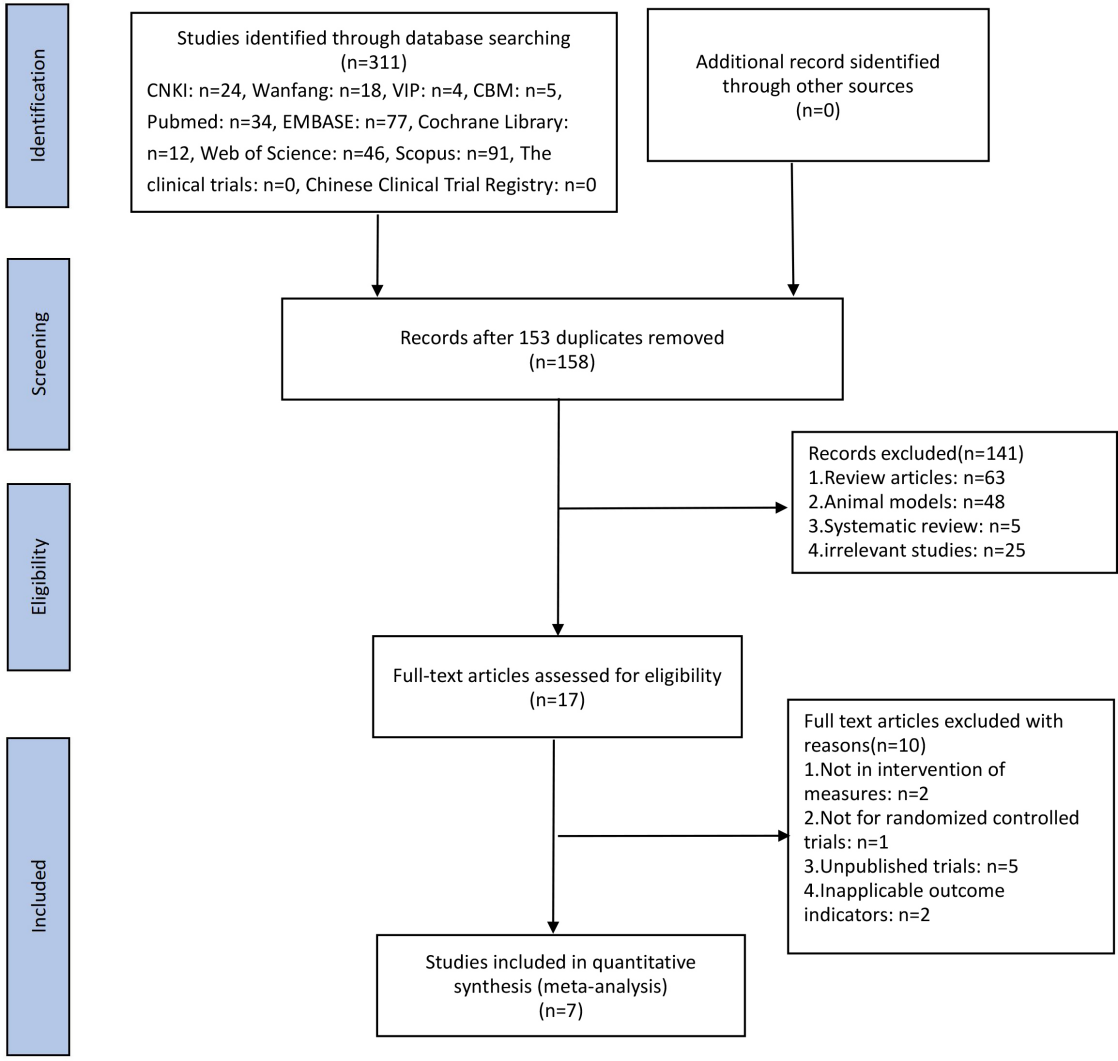
Flow diagram of the study selection process.

Three hundred and eleven potentially relevant papers were imported into NoteExpress after searches across databases. After removal of duplicates across databases and reviewing of titles and abstracts, 158 papers were deemed to be of potential interest for further consideration and full texts were retrieved. Review of the full text rapidly eliminated the majority of studies and in all 7 RCTs (26–29, 33–35) met the eligibility requirements and were included in the meta-analysis. The literature selection process is depicted in **Figure 1**.

### Study characteristics

The main characteristics of the included studies in the present meta-analysis are described in **Table 1**. Overall, a total of 447 participants in the 7 RCTs that were conducted in Iran, Turkey and China was analyzed. Among the 7 included studies, 5 studies compared curcumin/CL water decoction with placebo (26, 28, 29, 33, 34), 2 studies compared curcumin/CL water decoction plus metformin with metformin alone (27, 35). The dosage of curcumin ranged from 80 to 1500 mg/day and CL water decoction’s dosage was 90ml/day, the duration of the intervention varied from 6 weeks to 6 months. Primary outcome measures included fasting blood glucose (FBG), insulin (INS), homeostatic model assessment for insulin resistance (HOMA-IR) and C-reactive protein (CRP).

**Table 1:** The characteristics of the included studies.

Reference	Nation	Sample size(T/C)	Mean age (T/C)	Intervention	Comparison	Dosage(T/C)	Treatment duration	Outcomes	Adverse reaction
Jamilian M 2020 (26)	Iran	24/26	28.6 ± 4.7/27.2 ± 3.4	Curcumin	Placebo	Curcumin 500mg, qd/Placebo Nr	12 weeks	Weight, BMI, FBG, INS, QUICKI, HOMA-IR, T, DHEAS, LH, FSH, LH/FSH, TG, TC, LDL-C, HDL-C	Nr
Sohrevardi SM2021 (27)	Iran	48/50	29 ± 2/28.8 ± 2.46	Curcumin+Metformin	Metformin	Curcumin 80mg, qd Metformin 500mg, tid/Metformin 500mg, tid	12 weeks	Weight, BMI, FBG, INS, QUICKI, HOMA-IR, T, DHEAS, LH, FSH, LH/FSH, TG, TC, LDL-C, HDL-C	Nr
Sohaei S 2019 (29)	Iran	27/24	29.40 ± 5.33/29.58 ± 5	Curcumin	Placebo	Curcumin 500mg, bid/Placebo Nr	6 weeks	Weight, BMI, FBG, INS, QUICKI, HOMA-IR, TG, TC, LDL-C, HDL-C, CRP	Nr
Asan SA 2020 (33)	Turkey	15/15	27.6 ± 3.6/28.3 ± 5.9	Curcumin	Placebo	Curcumin 93.34mg/Placebo Nr	8 weeks	Weight, WC, BMI, FBG, INS, HOMA-IR, T, DHEAS, LH, FSH, TG, TC, LDL-C, HDL-C, CRP	Nr
Heshmati J 2021 (34)	Iran	34/33	30.97 ± 5.20/30.75 ± 7.97	Curcumin	Placebo	Curcumin 500mg, tid/Placebo 500mg, tid	12 weeks	WC, BMI, FBG, INS, QUICKI, HOMA-IR, DHEAS, LH, FSH	Nr
Wu JL 2022 (28)	China	47/44	27.06 ± 4.99/27.16 ± 4.87	CL water decoction	Placebo	CL water decoction 45ml, bid/Placebo 45ml, bid	6 months	BMI, WHR, FBG, INS, Glu120, Ins120, HbA1c, HOMA-IR, LH/FSH, FAI, RBC, WBC, Cr, ALT, AST	T: pruritus (n = 1),edema (n = 1), nausea (n = 1), dizzy (n = 1) C: none
Wu JL 2020 (35)	China	30/30	26.1 ± 4.9/25. 6 ± 5.0	CL water decoction+ Metformin	Metformin	CL water decoction 45ml, bid Metformin 0.85g, bid/Metformin 0.85g, bid	3 months	BMI, WHR, FBG, INS, Glu120, Ins120, HbA1c, HOMA-IR, LH/FSH, FAI, TG, TC, LDL-C, HDL-C, RBC, WBC, Cr, ALT, AST	T:nausea (n = 2), Bloating (n = 2), diarrhea (n = 2), constipation (n=3), dizzy (n = 2), pruritus (n = 5), edema (n= 1) C: nausea (n = 2), Bloating (n = 2), diarrhea (n = 3), constipation (n=1), dizzy (n = 1), pruritus (n = 3)

CL, Curcuma Longa; WC, waist circumference; BMI, body mass index; WHR, waist-to-hip ratio; FBG, fasting blood glucose; INS, insulin; QUICKI, quantitative insulin sensitivity check index; Glu120, Blood glucose at 2 h after OGTT; Ins120, Insulin at 2 h after OGTT; HbA1c, Glycosylated hemoglobin A1c; HOMA-IR, Homeostatic model assessment for insulin resistance; T, testosterone; DHEAS, dehydroepiandrosterone-sulfate; LH, luteinizing hormone; FSH, follicle-stimulating hormone; LH/FSH, luteinizing hormone/follicle-stimulating hormone; FAI, free androgen index; TG, triglycerides; TC, Total Cholesterol; LDL-C, low-density lipoprotein cholesterol; HDL-C, high-density lipoprotein cholesterol; CRP, C-reactive protein; RBC, red blood cell; WBC, white blood cell; Cr, Creatinine; ALT, alanine aminotransferase; AST, aspartate aminotransferase; Nr, not report; ±, operator symbol, values are expressed as mean ± SD.

### Risk-of-bias assessment

**Figure 2:**
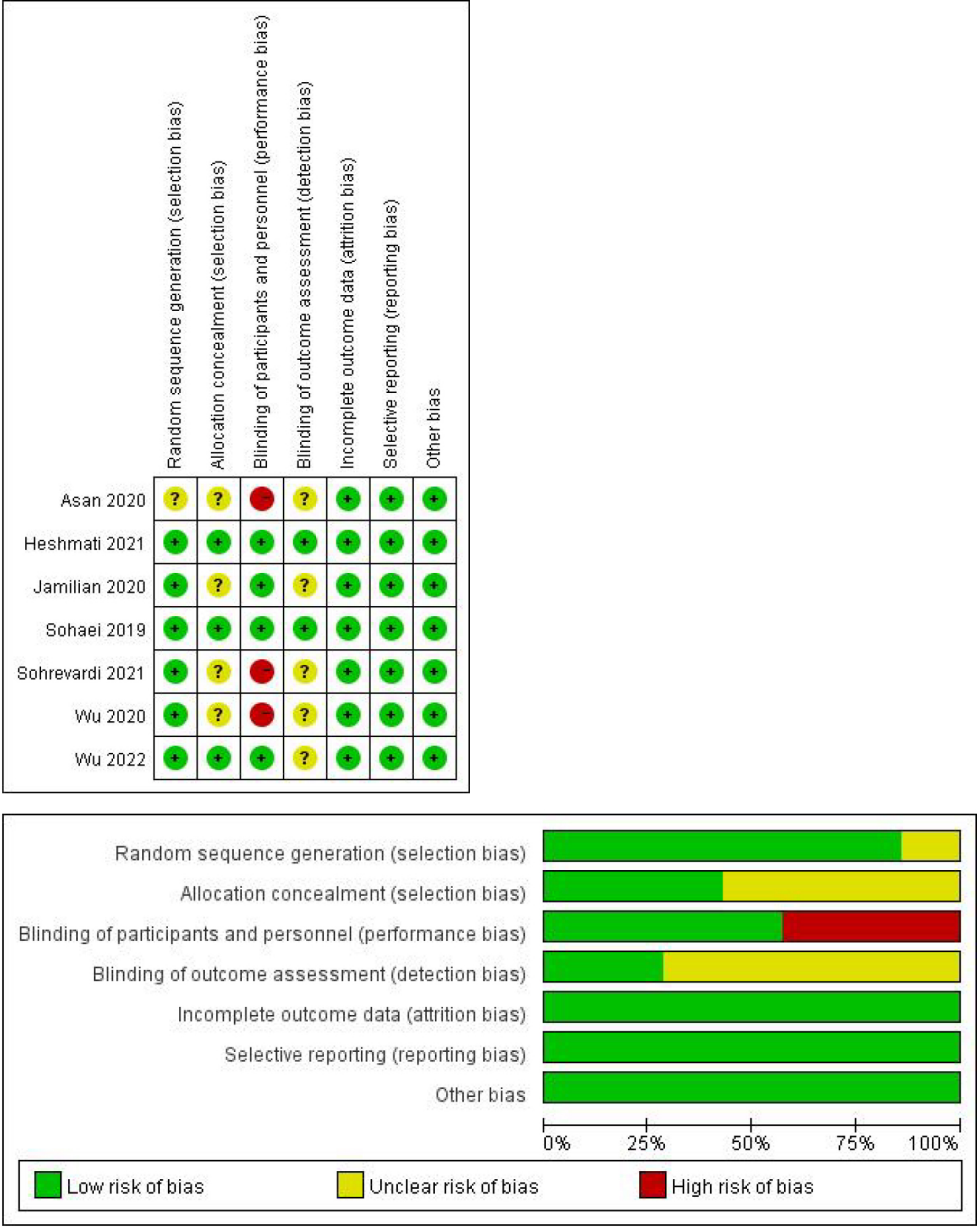
Risk of bias.

**Figure 2** summarizes the risk of bias of the included studies according to the pre-defined criteria in Cochrane handbook. Adequate randomized sequence generation was reported in all included trials except one (33). Most randomized trials did not report whether allocation was concealed (26, 27, 33, 35), and participants were not blinded to randomization 3 trials (27, 33, 35). 2 studies (29, 34) specified that the evaluators of outcome assessors were blinded and were given a low risk of bias. There was a low risk of bias of incomplete outcome data, selective reporting, and other sources in all studies. We did not assess funnel plots for publication bias because fewer than 10 studies were included in the meta-analysis.

## Outcome measures

### Effect of curcumin on anthropometric parameters

**Figure 3:**
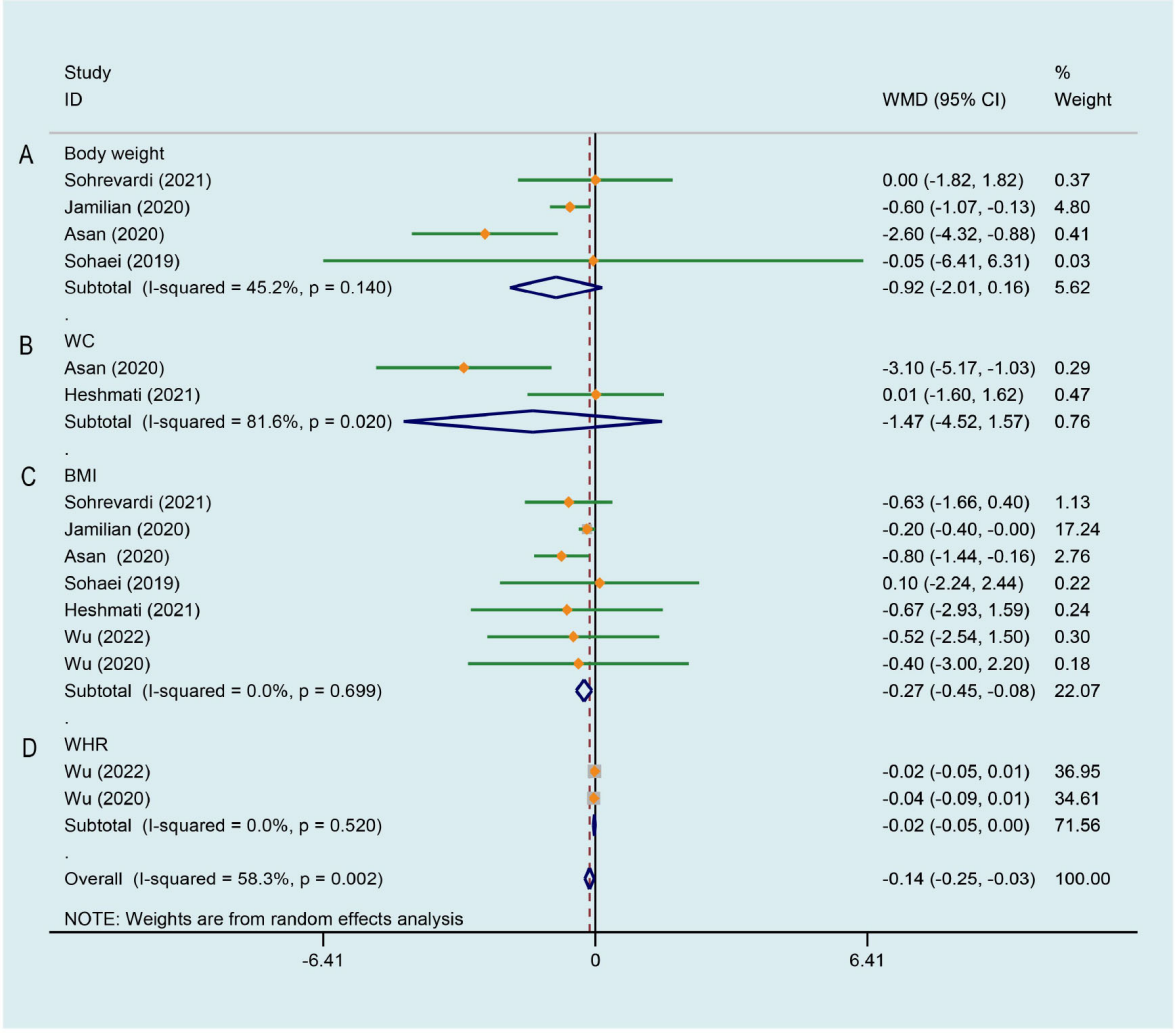
Meta-analyses of the effect of curcumin on anthropometric parameters. **(A)** Body weight, **(B)** WC, **(C)** BMI, **(D)** WHR.

4 studies (26, 27, 29, 33) with 229 patients were involved in this analysis. The result reported apparent trend for curcumin to decrease weight in PCOS patients and there was a medium degree of heterogeneity (WMD -0.924, 95% CI -2.009 to 0.162, P = 0.095, I^2^ = 45.2%, **Figure 3A**). Only two RCTs (33, 34) reported waist circumference (WC), there was no significant difference in WC of the intervention groups when compared with the placebo groups, and with high heterogeneity (WMD -1.475, 95% CI -4.519 to 1.570, P = 0.342, I^2^ = 81.6%, **Figure 3B**). 7 trials (26–29, 33–35) evaluated the effects of curcumin on body mass index (BMI) in this review, there were 225 patients in the intervention group and 222 in the control group. Meta-analysis revealed a significant BMI-lowering effect favoring the experimental group compared to the control group (WMD -0.267, 95% CI -0.450 to -0.084, P = 0.004, I^2^ = 0.0%, **Figure 3C**). 2 studies (28, 35) analyzed the effects of CL water decoction on waist-to-hip ratio (WHR) in PCOS patients. Compared with the control group, there was no significant difference in WHR in the intervention group (WMD -0.024, 95% CI -0.048 to 0.000, P = 0.052, I^2^ = 0.0%, **Figure 3D**).

### Effect of curcumin on CRP

**Figure 4:**
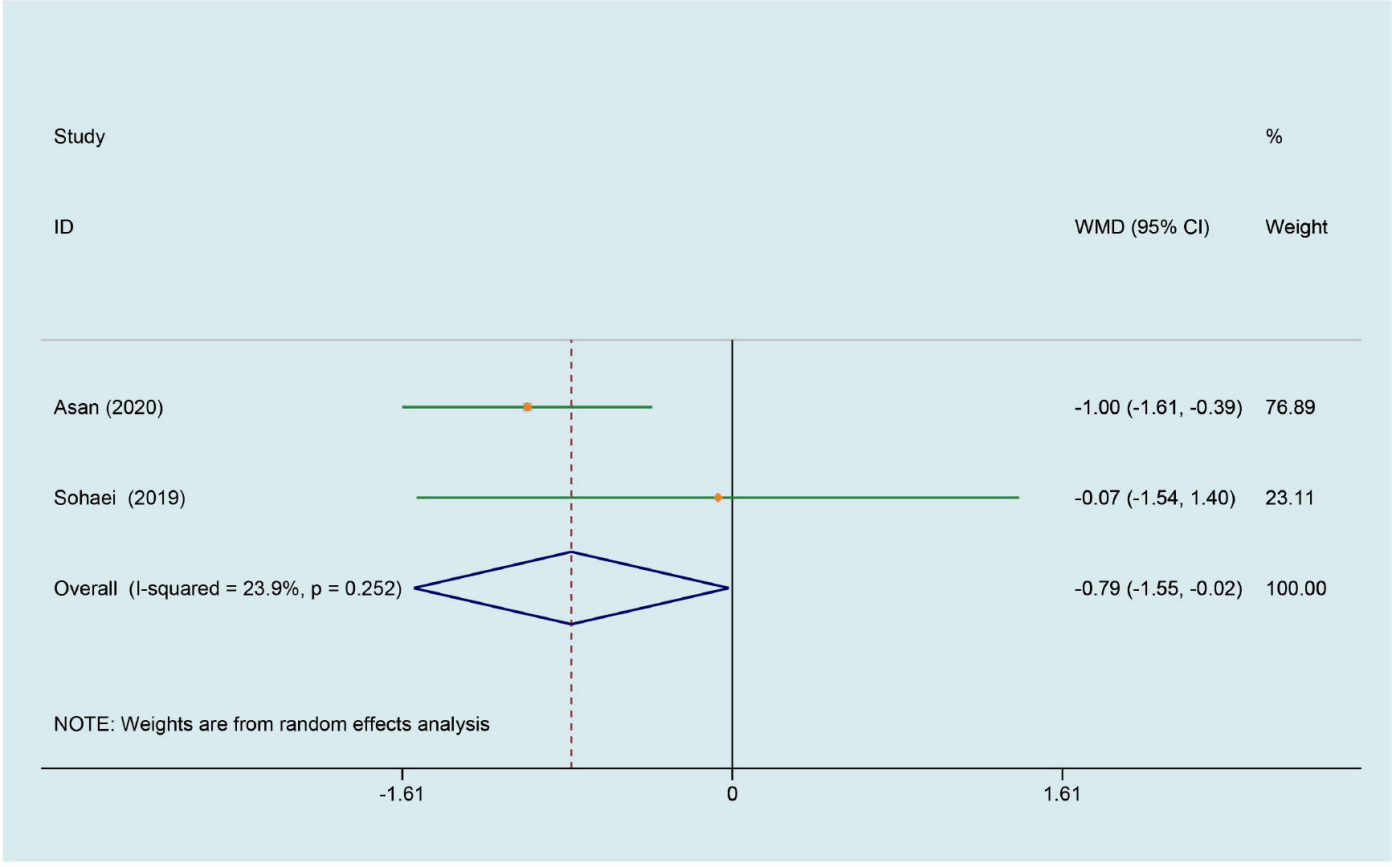
Meta-analyses of the effect of curcumin on CRP.

The level of CRP was evaluated in the 2 trials comparing curcumin with placebo (29, 33). The meta-analysis revealed a significant reduction by the treatment of curcumin (WMD -0.785, 95% CI -1.553 to -0.017, P = 0.045, I^2^ = 23.9%, **Figure 4**).

### Effect of curcumin on glucose metabolism

**Figure 5:**
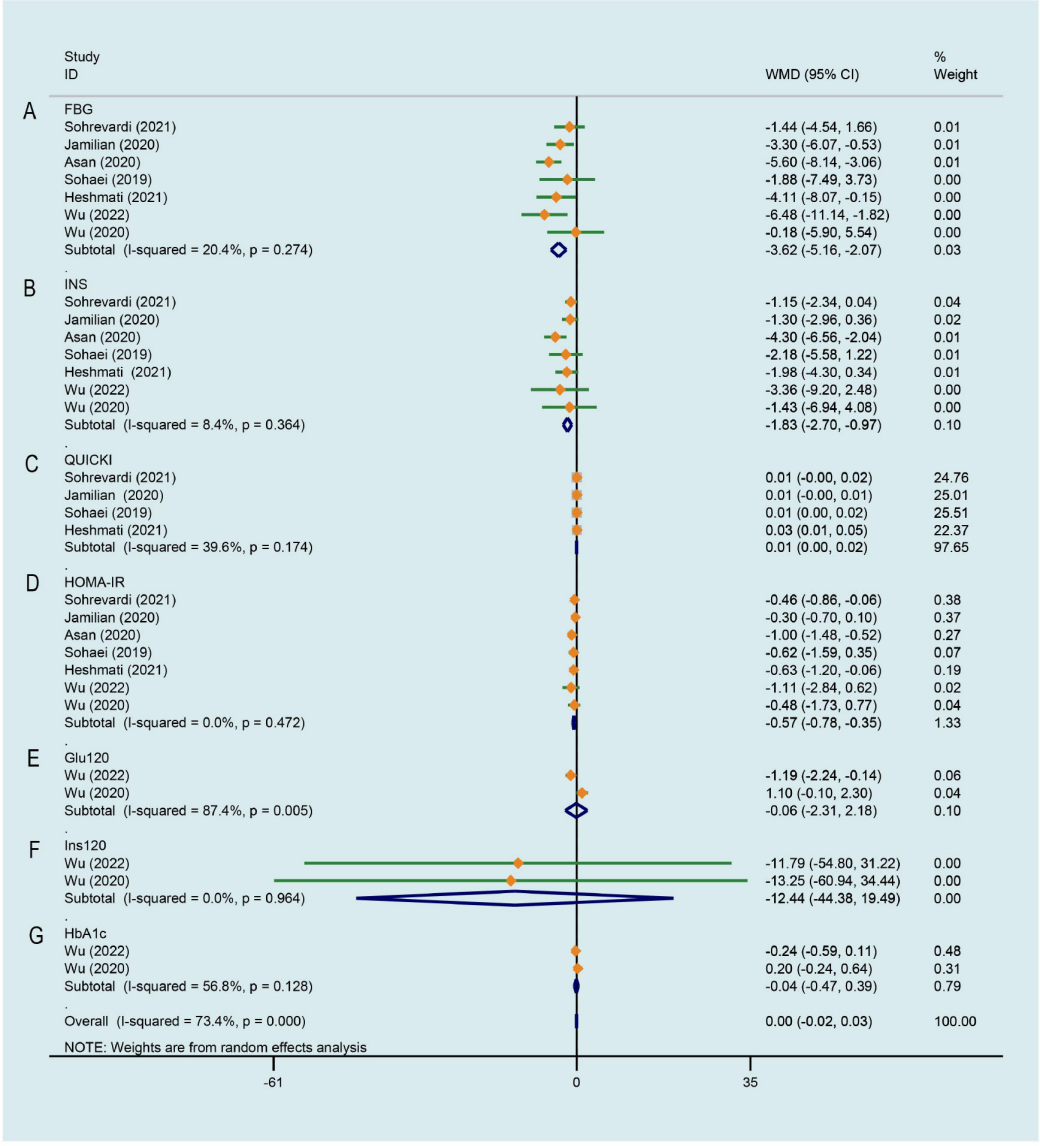
Meta-analyses of the effect of curcumin on glucose metabolism. **(A)** FBG, **(B)** INS, **(C)** QUICKI, **(D)** HOMA-IR, **(E)** Glu120, **(F)** Ins120, **(G)** HbA1c.

As illustrated in **Figure 5A**, a significant decrease of PCOS patients’ FBG was observed after curcumin treatment comparing to that of the control group, there was a low degree of heterogeneity across the study data (WMD -3.618, 95% CI -5.165 to -2.071, P < 0.001, I^2^ = 20.4%). **Figure 5B** displays the effects of curcumin on INS across 7 RCTs (26–29, 33–35), the study data has low heterogeneity. Compared with the control group, PCOS patients treated with curcumin/CL water decoction had significantly lower INS (WMD -1.834, 95% CI -2.701 to -0.968, P < 0.001, I^2^ = 8.4%). The effect of curcumin on quantitative insulin sensitivity check index (QUICKI) was evaluated in 4 studies (26, 27, 29, 34). Compared with the control condition, a significant improvement on QUICKI was observed by the experimental group (WMD 0.011, 95% CI 0.005 to 0.017, P < 0.001, I^2^ = 39.6%, **Figure 5C**). For HOMA-IR, 7 studies (26–29, 33–35) involving 447 subjects suggested a significant improvement effect by the treatment group compared with the control group (WMD -0.565, 95% CI -0.779 to -0.351, P < 0.001, I^2^ = 0.0%, **Figure 5D**). Blood glucose at 2 h after OGTT (Glu120) was evaluated in two studies (28, 35) that compared CL water decoction/CL water decoction plus metformin with placebo/metformin alone. There was not strong evidence that the treatment group had an effect on improving Glu120 because of no statistical difference (WMD -0.063, 95% CI -2.307 to 2.181, P = 0.956, I^2^ = 87.4%, **Figure 5E**). Meta-analysis of 2 studies (28, 35) involving 151 patients showed that no significant difference between the treatment group and the control group was identified on the level of insulin at 2 h after OGTT (Ins120) (WMD -12.445, 95% CI -44.384 to 19.494, P = 0.445, I^2^ = 0.0%, **Figure 5F**). As shown in **Figure 5G**, when the treatment group was compared with the control group, there was no significant difference in the level of glycosylated hemoglobin A1c (HbA1c) between the two groups (WMD -0.042, 95% CI -0.471 to 0.387, P = 0.849, I^2^ = 56.8%) (28, 35).

### Effect of curcumin on lipid metabolism

**Figure 6:**
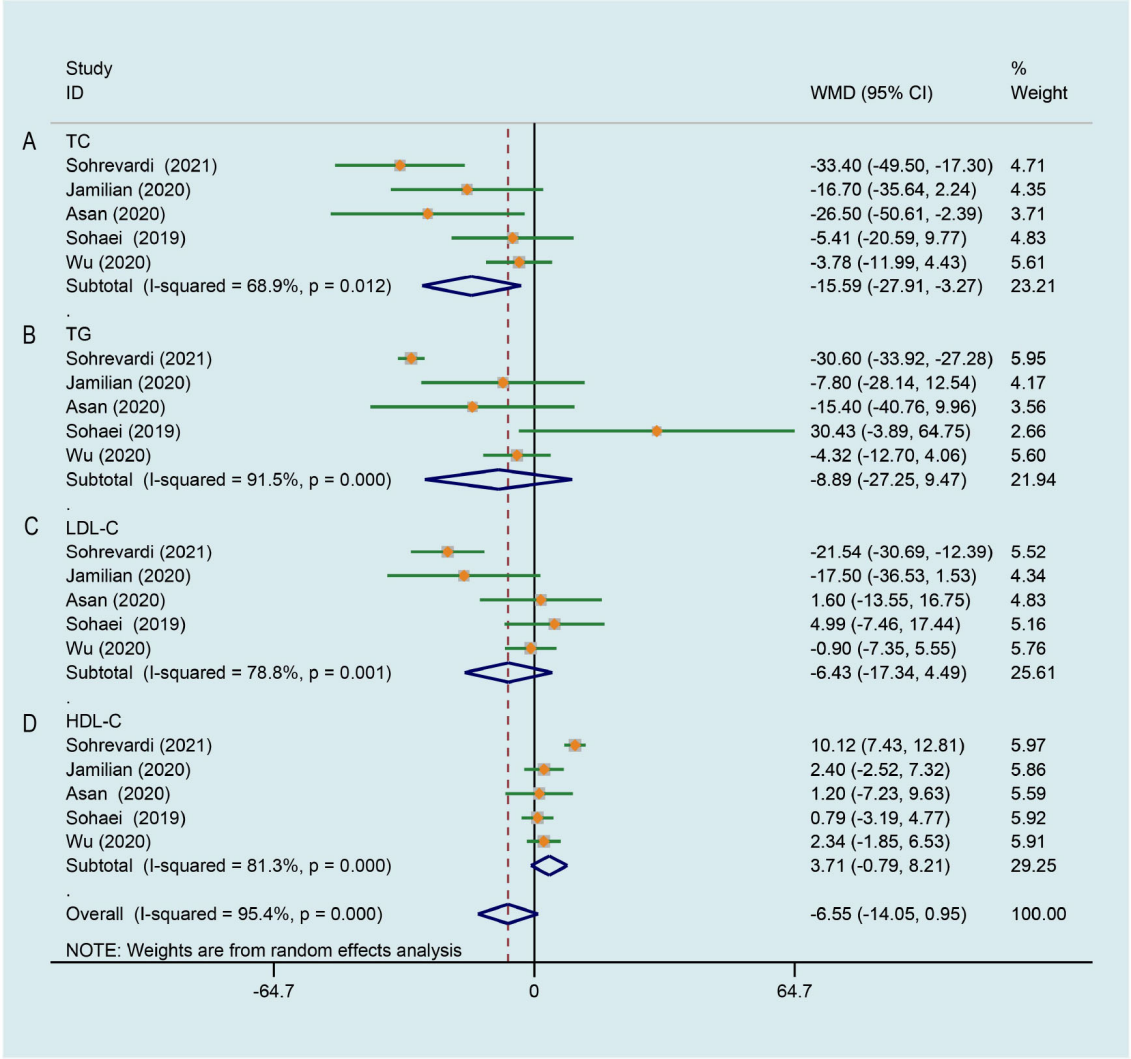
Meta-analyses of the effect of curcumin on lipid metabolism. **(A)** TC, **(B)** TG, **(C)** LDL-C, **(D)** HDL-C.

5 trials (26, 27, 29, 33, 35) evaluated the effects of curcumin on the level of total cholesterol (TC). Meta-analysis showed that curcumin/CL water decoction significantly decreased the level of TC in patients with PCOS (WMD -15.591, 95% CI -27.908 to -3.273, P = 0.013, I^2^ = 68.9%, **Figure 6A**). The whole five data (26, 27, 29, 33, 35) were pooled and significant improving effects of curcumin on triglycerides (TG) (WMD -8.889, 95% CI -27.246 to 9.468, P = 0.343, I^2^ = 91.5%, **Figure 6B**), low-density lipoprotein cholesterol (LDL-C) (WMD -6.427, 95% CI -17.343 to 4.489, P = 0.249, I^2^ = 78.8%, **Figure 6C**) and high-density lipoprotein cholesterol (HDL-C) (WMD 3.713, 95% CI -0.786 to 8.211, P = 0.106, I^2^ = 81.3%, **Figure 6D**) were not identified compared to the control group. The heterogeneities in the study data of TG, LDL-C and HDL-C were all high.

### Effect of curcumin on hormone parameters

**Figure 7:**
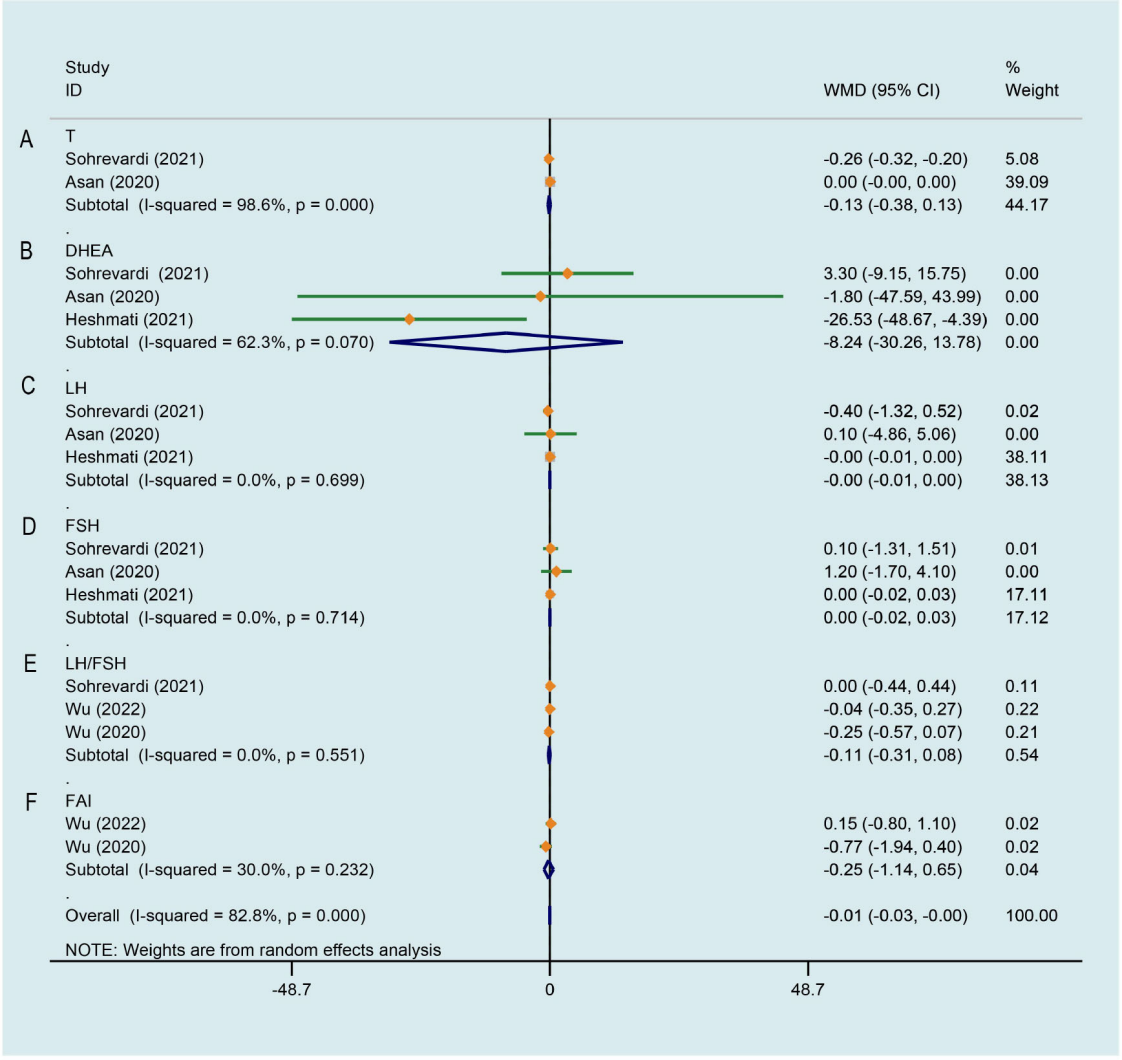
Meta-analyses of the effect of curcumin on hormone parameters. **(A)** T, **(B)** DHEA, **(C)** LH, **(D)** FSH, **(E)** LH/FSH, **(F)** FAI.

There was significant heterogeneity across the study data, and our result revealed curcumin had no significant effect on improving testosterone (T) level of PCOS patients in comparison with the control group (WMD -0.128, 95% CI -0.383 to 0.127, P = 0.326, I^2^ = 98.6%, **Figure 7A**). Random effects meta-analysis found no significant effect for curcumin reducing level of dehydroepiandrosterone-sulfate (DHEA) in comparison with the control group (WMD -8.239, 95% CI -30.260 to 13.781, P = 0.463, I^2^ = 62.3%, **Figure 7B**). As shown in **Figures 7C, D**, pooling 3 RCTs (27, 33, 34) together did not show any significant change in luteinizing hormone (LH) (WMD -0.003, 95% CI -0.007 to 0.000, P = 0.087, I^2^ = 0.0%) and follicle-stimulating hormone (FSH) (WMD 0.002, 95% CI -0.024 to 0.029, P = 0.854, I^2^ = 0.0%) of PCOS patients after curcumin treatment comparing to that of control group. Compared to the control group, an evident improvement on LH/FSH was not observed by curcumin in 3 studies (WMD -0.114, 95% CI -0.311 to 0.084, P = 0.259, I^2^ = 0.0%, **Figure 7E**) (27, 28, 35). In terms of ameliorating free androgen index (FAI), there was not a significant difference between the intervention group and the control group (WMD -0.245, 95% CI -1.138 to 0.647, P = 0.590, I^2^ = 30.0%, **Figure 7F**).

### Adverse events

**Figure 8:**
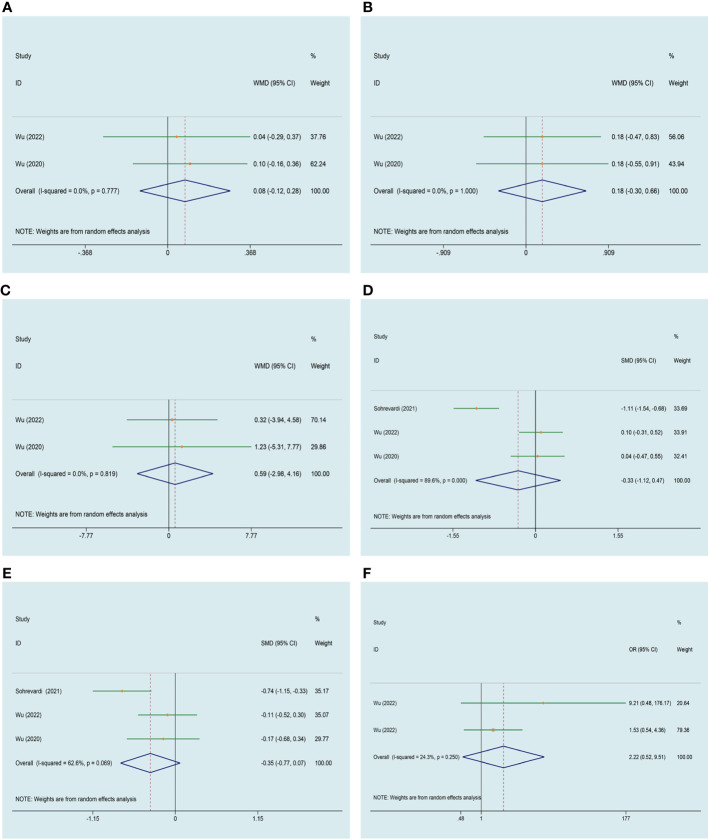
Meta-analyses of the effect of curcumin on adverse events. **(A)** RBC, **(B)** WBC, **(C)** Cr, **(D)** ALT, **(E)** AST, **(F)** adverse events.

2 studies (28, 35) were included that evaluated the influence of curcumin on red blood cell (RBC), white blood cell (WBC) and creatinine (Cr). Meta-analyses found no obvious improvement on the level of RBC (WMD 0.077, 95% CI -0.124 to 0.279, P = 0.452, I^2^ = 0.0%, **Figure 8A**), WBC (WMD 0.180, 95% CI -0.303 to 0.663, P = 0.465, I^2^ = 0.0%, **Figure 8B**) and Cr (WMD 0.592, 95% CI -2.980 to 4.163, P = 0.745, I^2^ = 0.0%, **Figure 8C**) in PCOS women after treatment with curcumin versus the comparison group. Meta-analysis of three studies (27, 28, 35) assessed the effect of curcumin on alanine aminotransferase (ALT) and aspartate aminotransferase (AST), there was no significant difference in ALT (SMD -0.325, 95% CI -1.124 to 0.473, P = 0.424, I^2^ = 89.6%, **Figure 8D**) and AST (SMD -0.350, 95% CI -0.766 to 0.066, P = 0.099, I^2^ = 62.6%, **Figure 8E**) between the groups. Two of the enrolled studies included adverse events, the meta-analysis showed that it was not more possible for curcumin to cause adverse events, it may be a safe therapeutic method (OR 2.215, 95% CI 0.516 to 9.512, P = 0.285, I^2^ = 24.3%, **Figure 8F**).

### Sensitivity analysis

Based on the results of our meta-analysis, we performed a sensitivity analysis for outcomes with high heterogeneity: WC, Glu 120, HbA1c, T, DHEA, TG, TC, LDL-C, HDL-C, ALT and AST. The results of sensitivity analyses showed that all the points fell in the confidence interval, indicating that none of the individual studies affected the final conclusion obviously (**Supplementary Appendix 2**).

## Discussion

To the best of our knowledge, this is currently the most comprehensive systematic review and meta-analysis of the effect of curcumin specifically for patients undergoing treatment for PCOS, in which we systematically searched and analyzed results from 7 eligible RCTs that involved 447 participants with PCOS. This analysis found that curcumin can significantly ameliorate HOMA-IR, FBG, INS, QUICKI and TC when compared with control group. In contrast to previous meta-analyses, curcumin also has a significant large positive effect size in CRP and BMI. Furthermore, we have also found that significantly decreasing trends of weight, LH and WHR after the curcumin intervention. As for the safety, curcumin appears to be well-tolerated with few adverse events reported by the included studies. However, this meta-analysis included a limited number of high-quality studies, therefore more longer-term and large sample trials evaluating the efficacy and safety of curcumin for PCOS are warranted.

### Curcumin and anthropometric parameters

Women with PCOS report significant concern regarding weight gain, the rates of weight gain can be higher, which is more likely to be obese (36). Obesity is related to the infertility of PCOS and is a major risk factor for type 2 diabetes and cardiovascular disease in women (37, 38). In view of evidence-based guidelines on PCOS treatment, lifestyle management, including diet, exercise and behavioral strategies, is the first-line management in the intervention hierarchy in PCOS (39). In recent years, functional foods and nutraceuticals which have been shown as potential secondary therapies for the prevention of cardiovascular risk factors have been proposed for the prevention against chronic diseases, glycemic and lipid metabolic disorders, and multiple metabolic syndrome components (40, 41). The results of our meta-analysis might confirm curcumin’s effects on body composition indices. Our results highlighted an overall reduction in the level of BMI as a result of curcumin. This finding was in agreement with studies from a previous meta-analysis of 11 studies in which curcumin intervention significantly decreased the level of BMI in patients with overweight or obese (42). Several previous studies have reported the mechanisms that curcumin might affect body composition indices: curcumin can affect certain signal transduction and regulate the expression of specific cytokines (such as interleukin-1β, interleukin-6 (IL-6), TNF-α, monocyte chemoattractant protein-1, leptin and adiponectin), thereby maintaining energy homeostasis (43, 44). On the other hand, curcumin also induces the conversion of white adipocytes to a brown fat phenotype (45), which facilitates energy metabolism.

### Curcumin and CRP

Previous studies have demonstrated that PCOS-related metabolic diseases, such as insulin resistance, obesity, type 2 diabetes and atherosclerosis are linked to chronic low-grade inflammation (46). In addition, pro‐inflammatory factor can also promote the proliferation of ovarian granulosa cell and ovarian follicular membrane cells to produce more androgen leading to hyperandrogenemia (47). A number of studies have confirmed the anti-inflammatory properties of curcumin on PCOS in clinical research and animal models. Mohammadi et al. (48) found that the number of necrotic cells, IR index and IL-6 levels in adult female Wistar rats with PCOS were significantly reduced after curcumin treatment. Sohaei et al. (29) also observed, after curcumin therapy, a significant improvement in CRP after treatment of 27 patients with PCOS, which was consistent with the results of our studies. CRP is one of the members of the pentraxin family in hepatocytes, whose expression is mainly activated by IL-6 and regulated by nuclear factor kappa-light-chain-enhancer of activated B cells (NF-kB) signal path (49). Curcumin exhibits potent anti-inflammatory activity *via* suppression of IkB kinase activity and NF-kB signaling pathway (50).

### Curcumin and glycolipid metabolism

The prevalence of metabolic syndrome (MS) among PCOS patients has been reported to be about 2 times higher compared to that in the general population (51). Both insulin resistance and dyslipidemia are associated with metabolic disorder in PCOS patients, which have been evidenced as risk factors for T2DM and cardiovascular diseases (52, 53). Curcumin has been widely investigated owing to its obvious effects on improving glucose metabolism and lowering blood lipids. Compared with the control group, it was observed a substantial decrease in FBG, INS and HOMA-IR and a marked increase in QUICKI of PCOS patients in this meta-analysis. Hypoglycemic properties of curcumin have been known since 1972 (54), the action is probably mediated by the stimulation of the PI3K/Akt pathway, which in turn promotes the translocation of glucose transporter 4 (GLUT4) to the plasma membrane, leading to an increase in glucose uptake and glycolysis (55). In the study by Wu et al (35), a remarkable rise of disposition index and glucose disposal rate has been observed after taking curcumin for 3 months, suggesting that curcumin ameliorates glucose homeostasis through protection of islet B cells. Recently, the importance of postprandial hyperglycemia has been highlighted by the fact that uncontrolled postprandial hyperglycemia gradually causes pancreatic β-cell exhaustion (56, 57). Furthermore, it has been shown that fluctuating glucose also produces oxidative stress, thereby inducing endothelial dysfunction and inflammation (56). A study in experimental animals has demonstrated that curcumin treatment for 8 weeks decreases both postprandial glycemia and HbA1c (58), which contradicted our findings. Nevertheless, we cannot deny the positive effect of curcumin on the postprandial glucose control of PCOS individuals, due to the limited number of studies. Possible mechanisms for the hypolipidemic effect of curcumin could involve increasing polyunsaturated sphingomyelin expression, improving the apoptotic status of liver tissue and inhibiting oxidative stress *via* downregulating malondialdehyde (MDA) levels and upregulating superoxide dismutase (SOD) levels (59, 60). But in our result, we did not find significant effects of curcumin on blood lipids (HDL-C, TG and LDL-C) other than TC. These inconsistent results may be ascribed, at least in part, to differences in study population, doses of curcumin and analytical approaches. The results, therefore, need to be interpreted with caution and larger studies are required to validate the results.

### Curcumin and sex hormone

Hyperandrogenism is implicated as a key mediator of the pathogenesis of PCOS, which persists throughout reproductive life (61). The pathogenesis may include abnormal gonadotropin secretion and hyperinsulinism caused by IR. Abnormally increased LH pulse frequency and amplitude further enhance androgen synthesis in ovarian theca cell and promote hyperandrogenemia in patients with PCOS (62). Hyperinsulinemia may cause an augmented androgen production in the adrenal cortex and follicles *via* stimulation of LH secretion and a decreased SHBG production, resulting elevated androgen levels that may lead to the characteristic clinical manifestations like acne and hirsutism (63). The present meta-analysis has not demonstrated that curcumin has good efficacy on female reproductive hormones, however, several studies provided strong justification for further exploration. A study by Heshmati et al (34), investigating the effect of curcumin on patients with PCOS, showed a significant reduction in DHEA after the curcumin than placebo. In another study, the experimental group of women that were diagnosed with PCOS, following the treatment with curcumin, manifested a clear descending trend of the levels of FAI (35). From these, curcumin has potential effects on lowering androgen levels in patients with PCOS. Most of the analysis results of our research are negative, but we cannot exclude that curcumin might be playing an active role in various reproductive hormones of PCOS patients.

### Adverse effects

No serious side effects occurred as a result in our study, and only a small number of patients complained of minor side effects such as mild gastrointestinal discomfort and pruritus. Simultaneously, we observed that biochemical parameters such as RBC, WBC, Cr, AST and ALT did not show any gross abnormalities in expression, indicating that there was no obvious damage to the blood routine and liver and kidney function, which is one of the advantages of this meta-analysis. Moreover, a randomized, double-blind, placebo-controlled clinical trial found that when the clinical dose was 2400mg/d, curcumin supplementation could reduce systolic blood pressure and had no effect on cardiac metabolic risk parameters (64). In the United States, curcumin is approved as safe by the Food and Drug Administration (FDA) (65). From the current evidence, curcumin seems to be generally well tolerated and safe, although more clinical studies are needed to confirm the safety of curcumin in long-term treatment.

### Strengths and limitations

To the best of our understanding, compared with the previously published results, this study is the first meta-analysis of RCTs to simultaneously evaluate the effects of anthropometric indicators, glucose and lipid metabolism, inflammatory factors, sex hormone levels and adverse reactions in PCOS, and provides evidence for curcumin as a non-toxic and safe drug to treat PCOS. In addition, all tests included in our analysis are clearly based on the Rotterdam standard, which is highly homogeneous. However, our review has several important limitations that need to be recognized. First, the limited sample size of the meta-analysis (a total of 447 randomized patients) resulted in weak evidence-based conclusion of the effectiveness of curcumin. Second, the descriptions of the allocation concealment or blinding were sparse in most of the included trials, which may lead to performance bias in outcome measurement. As such, these findings should be treated with caution until replicated. In addition, the duration of the involved studies was generally short-to-medium term (mostly 6 weeks to 3 months), and there was a lack of follow-up observation on the long-term efficacy of curcumin. Finally, most randomized controlled trials came from the Middle East (mainly Iran and Turkey) and the Asia Pacific region (especially East Asia, such as China), and there were no eligible studies from Western Europe and North America. Therefore, the representativeness of research results has some limitations. Collectively, there is an absence of more racially and ethnically high-quality data in our study. At present, we cannot provide robust support for the efficacy and safety of curcumin in treating PCOS, but it will lay the foundation for future large-scale trials.

### Implications for future

More strictly designed studies are needed to confirm the impact of curcumin on PCOS, and large sample, longer-term multi center, high-quality and well-designed clinical trials should be registered to better understand the potential mechanism of curcumin’s efficacy on patients with PCOS and provide decision-making for clinical evidence-based treatment. In addition, research that includes patient data from other countries or regions in the world will help to expand the applicability of the results.

## Conclusion

Altogether, the results of this meta-analysis are inspiring and provide evidence supporting the potential effectiveness and safety of curcumin in orchestrating the inflammatory microenvironment and reducing the risk of abnormalities of glucose and lipid metabolism and obesity in patients with PCOS. However, the strength of this conclusion is tempered by the dearth of large-scale, high-quality reference datasets and the significant number of studies on this topic. Indeed, the effect sizes reported in this analysis merit further evaluation in a larger, well-designed, high-quality prospective randomized clinical trial. Studies that explore the different doses and types of the supplement are also required for access to high solubility and bioavailability curcumin.

## Data availability statement

The original contributions presented in the study are included in the article/**Supplementary Material**. Further inquiries can be directed to the corresponding author.

## Author contributions

WJS and YZ conceptualized the research question. YFQ and HJ participated in drafting and writing the review. YFQ, HWW, and HJ participated in the formulation of retrieval strategies, data acquisition, data analysis and quality assessment. YJP and YHZ participated in the drawing of tables and figures. XKW and YHH participated in critical revision of the manuscript. All authors contributed to the research and approved the final manuscript.

## Funding

This work is supported by the Young Scientists Project of the National Natural Science Foundation of China (81803945), National Natural Science Foundation of China (82074259), Scientific Research Project of Traditional Chinese Medicine in Heilongjiang Province (ZHY19024), the Project of Young Innovative Talents in Colleges and Universities in Heilongjiang Province (UNPYSCT-2016216), and Heilongjiang University of Traditional Chinese Medicine Graduate innovation research project (2022yjscx017).

## Conflict of interest

The authors declare that the research was conducted in the absence of any commercial or financial relationships that could be construed as a potential conflict of interest.

## Publisher’s note

All claims expressed in this article are solely those of the authors and do not necessarily represent those of their affiliated organizations, or those of the publisher, the editors and the reviewers. Any product that may be evaluated in this article, or claim that may be made by its manufacturer, is not guaranteed or endorsed by the publisher.
