# Supplementary material for: Therapeutic effect and safety of curcumin in women with PCOS: A systematic review and meta-analysis
Source: Front Endocrinol (Lausanne). 2022 Oct 27;13:1051111. doi: 10.3389/fendo.2022.1051111 (PMC9646792; doi:10.3389/fendo.2022.1051111)
Supplement: Supplementary file 1 [file DataSheet_1.pdf]

Online Appendix 1 Details of Search Strategy Source: PubMed; Searched on: May 2022; Results: 34

Search	Query
#1	“Polycystic Ovary Syndrome”[Mesh]
#2	Polycystic Ovary Syndrome[Title/Abstract]
#3	Ovary Syndrome, Polycystic[Title/Abstract]
#4	Syndrome, Polycystic Ovary[Title/Abstract]
#5	Stein-Leventhal Syndrome[Title/Abstract]
#6	Stein Leventhal Syndrome[Title/Abstract]
#7	Syndrome, Stein-Leventhal[Title/Abstract]
#8	Sclerocystic Ovary Syndrome[Title/Abstract]
#9	Ovarian Degeneration, Sclerocystic[Title/Abstract]
#10	Sclerocystic Ovary Syndrome[Title/Abstract]
#11	Polycystic Ovarian Syndrome[Title/Abstract]
#12	Ovarian Syndrome, Polycystic[Title/Abstract]
#13	Polycystic Ovary Syndrome 1[Title/Abstract]
#14	Sclerocystic Ovaries[Title/Abstract]
#15	Ovary, Sclerocystic[Title/Abstract]
#16	Sclerocystic Ovary[Title/Abstract]
#17	ovary polycystic disease[Title/Abstract]
#18	polycystic ovary[Title/Abstract]
#19	PCO[Title/Abstract]
#20	PCOS[Title/Abstract]
#21	#1 OR #2 OR #3 OR #4 OR #5 OR #6 OR #7 OR #8 OR #9 OR #10 OR #11 OR #12 OR #13 OR #14 OR #15 OR #16 OR #17 OR #18 OR #19 OR #20
#22	“Curcumin”[Mesh]
#23	Curcumin[Title/Abstract]
#24	nanocurcumin[Title/Abstract]
#25	Theracurmin[Title/Abstract]
#26	cur-cuminoid[Title/Abstract]
#27	Curcuminoid[Title/Abstract]
#28	Curcuminoids[Title/Abstract]
#29	curqfen[Title/Abstract]
#30	Turmeric Yellow[Title/Abstract]
#31	1,6-Heptadiene-3,5-dione, 1,7-bis(4-hydroxy-3-methoxyphenyl)-, (E,E)-[Title/Abstract]
#32	Yellow, Turmeric[Title/Abstract]
#33	Curcumin Phytosome[Title/Abstract]
#34	Phytosome, Curcumin[Title/Abstract]
#35	Diferuloylmethane[Title/Abstract]
#36	Mervia[Title/Abstract]
#37	Curcuma[Title/Abstract]
#38	Curcumas[Title/Abstract]
#39	Curcuma zedoaria[Title/Abstract]

#40	Curcuma zedoarias[Title/Abstract]
#41	curcumalonga[Title/Abstract]
#42	zedoaria, Curcuma[Title/Abstract]
#43	Zedoary zedoaria[Title/Abstract]
#44	Zedoary zedoarias[Title/Abstract]
#45	zedoaria, Zedoary[Title/Abstract]
#46	Curcuma longa[Title/Abstract]
#47	Curcuma longas[Title/Abstract]
#48	longa, Curcuma[Title/Abstract]
#49	Tumeric[Title/Abstract]
#50	Tumerics[Title/Abstract]
#51	Turmeric[Title/Abstract]
#52	Turmerics[Title/Abstract]
#53	#22 OR #23 OR #24 OR #25 OR #26 OR #27 OR #28 OR #29 OR #30 OR #31 OR #32 OR #33 OR #34 OR #35 OR #36 OR #37 OR #38 OR #39 OR #40 OR #41 OR #42 OR #43 OR #44 OR #45 OR #46 OR #47 OR #48 OR #49 OR #50 OR #51 OR #52
#54	#21 AND #53

Source: Cochrane Library; Searched on: May 2022; Results: 12

**Search**	**Query**
#1	MeSH descriptor: [Polycystic Ovary Syndrome] explode all trees
#2	(Polycystic Ovary Syndrome):ti,ab,kw
#3	(Ovary Syndrome, Polycystic):ti,ab,kw
#4	(Syndrome, Polycystic Ovary):ti,ab,kw
#5	(Stein-Leventhal Syndrome):ti,ab,kw
#6	(Stein Leventhal Syndrome):ti,ab,kw
#7	(Syndrome, Stein-Leventhal):ti,ab,kw
#8	(Sclerocystic Ovary Syndrome):ti,ab,kw
#9	(Ovarian Degeneration, Sclerocystic):ti,ab,kw
#10	(Sclerocystic Ovary Syndrome):ti,ab,kw
#11	(Polycystic Ovarian Syndrome):ti,ab,kw
#12	(Ovarian Syndrome, Polycystic):ti,ab,kw
#13	(Polycystic Ovary Syndrome 1):ti,ab,kw
#14	(Sclerocystic Ovaries):ti,ab,kw
#15	(Ovary, Sclerocystic):ti,ab,kw
#16	(Sclerocystic Ovary):ti,ab,kw
#17	(ovary polycystic disease):ti,ab,kw
#18	(polycystic ovary):ti,ab,kw
#19	(PCO):ti,ab,kw
#20	(PCOS):ti,ab,kw
#21	#1 OR #2 OR #3 OR #4 OR #5 OR #6 OR #7 OR #8 OR #9 OR #10 OR #11 OR #12 OR #13 OR #14 OR #15 OR #16 OR #17 OR #18 OR #19 OR #20
#22	MeSH descriptor: [Curcumin] explode all trees

#23	(Curcumin):ti,ab,kw
#24	(nanocurcumin):ti,ab,kw
#25	(Theracurmin):ti,ab,kw
#26	(cur-cuminoid):ti,ab,kw
#27	(Curcuminoid):ti,ab,kw
#28	(Curcuminoids):ti,ab,kw
#29	(curqfen):ti,ab,kw
#30	(Turmeric Yellow):ti,ab,kw
#31	(1,6-Heptadiene-3,5-dione, 1,7-bis(4-hydroxy-3-methoxyphenyl)-, (E,E)-):ti,ab,kw
#32	(Yellow, Turmeric):ti,ab,kw
#33	(Curcumin Phytosome):ti,ab,kw
#34	(Phytosome, Curcumin):ti,ab,kw
#35	(Diferuloylmethane):ti,ab,kw
#36	(Mervia):ti,ab,kw
#37	(Curcuma):ti,ab,kw
#38	(Curcumas):ti,ab,kw
#39	(Curcuma zedoaria):ti,ab,kw
#40	(Curcuma zedoarias):ti,ab,kw
#41	(curcumalonga):ti,ab,kw
#42	(zedoaria, Curcuma):ti,ab,kw
#43	(Zedoary zedoaria):ti,ab,kw
#44	(Zedoary zedoarias):ti,ab,kw
#45	(zedoaria, Zedoary):ti,ab,kw
#46	(Curcuma longa):ti,ab,kw
#47	(Curcuma longas):ti,ab,kw
#48	(longa, Curcuma):ti,ab,kw
#49	(Tumeric):ti,ab,kw
#50	(Tumerics):ti,ab,kw
#51	(Turmeric):ti,ab,kw
#52	(Turmerics):ti,ab,kw
#53	#22 OR #23 OR #24 OR #25 OR #26 OR #27 OR #28 OR #29 OR #30 OR #31 OR #32 OR #33 OR #34 OR #35 OR #36 OR #37 OR #38 OR #39 OR #40 OR #41 OR #42 OR #43 OR #44 OR #45 OR #46 OR #47 OR #48 OR #49 OR #50 OR #51 OR #52
#54	#21 AND #53

Source: Embase; Searched on: May 2022; Results: 77

**Search**	**Query**
#1	'ovary polycystic disease'/exp
#2	'ovary polycystic disease':ab,ti
#3	'Ovary Syndrome, Polycystic':ab,ti
#4	'Syndrome, Polycystic Ovary':ab,ti
#5	'Stein-Leventhal Syndrome':ab,ti
#6	'Stein Leventhal Syndrome':ab,ti

#7	'Syndrome, Stein-Leventhal':ab,ti
#8	'Sclerocystic Ovary Syndrome':ab,ti
#9	'Ovarian Degeneration, Sclerocystic':ab,ti
#10	'Sclerocystic Ovary Syndrome':ab,ti
#11	'Polycystic Ovarian Syndrome':ab,ti
#12	'Ovarian Syndrome, Polycystic':ab,ti
#13	'Polycystic Ovary Syndrome 1':ab,ti
#14	'Sclerocystic Ovaries':ab,ti
#15	'Ovary, Sclerocystic':ab,ti
#16	'Sclerocystic Ovary':ab,ti
#17	'Polycystic ovary syndrome':ab,ti
#18	'polycystic ovary':ab,ti
#19	'PCO':ab,ti
#20	'PCOS':ab,ti
#21	#1 OR #2 OR #3 OR #4 OR #5 OR #6 OR #7 OR #8 OR #9 OR #10 OR #11 OR #12 OR #13 OR #14 OR #15 OR #16 OR #17 OR #18 OR #19 OR #20
#22	'Curcumin'/exp
#23	'Curcumin':ab,ti
#24	'nanocurcumin':ab,ti
#25	'Theracurmin':ab,ti
#26	'cur-cuminoid':ab,ti
#27	'Curcuminoid':ab,ti
#28	'Curcuminoids':ab,ti
#29	'curqfen':ab,ti
#30	'Turmeric Yellow':ab,ti
#31	'1,6-Heptadiene-3,5-dione, 1,7-bis(4-hydroxy-3-methoxyphenyl)-, (E,E)-':ab,ti
#32	'Yellow, Turmeric':ab,ti
#33	'Curcumin Phytosome':ab,ti
#34	'Phytosome, Curcumin':ab,ti
#35	'Diferuloylmethane':ab,ti
#36	'Mervia':ab,ti
#37	'Curcuma':ab,ti
#38	'Curcumas':ab,ti
#39	'Curcuma zedoaria':ab,ti
#40	'Curcuma zedoarias':ab,ti
#41	'curcumalonga':ab,ti
#42	'zedoaria, Curcuma':ab,ti
#43	'Zedoary zedoaria':ab,ti
#44	'Zedoary zedoarias':ab,ti
#45	'zedoaria, Zedoary':ab,ti
#46	'Curcuma longa':ab,ti
#47	'Curcuma longas':ab,ti
#48	'longa, Curcuma':ab,ti

#49	'Tumeric':ab,ti
#50	'Tumerics':ab,ti
#51	'Turmeric':ab,ti
#52	'Turmerics':ab,ti
#53	#22 OR #23 OR #24 OR #25 OR #26 OR #27 OR #28 OR #29 OR #30 OR #31 OR #32 OR #33 OR #34 OR #35 OR #36 OR #37 OR #38 OR #39 OR #40 OR #41 OR #42 OR #43 OR #44 OR #45 OR #46 OR #47 OR #48 OR #49 OR #50 OR #51 OR #52
#54	#21 AND #53

Source: Scopus; Searched on: May 2022; Results: 91

Search	Query
#1	TITLE-ABS-KEY (polycystic AND ovary AND syndrome)
#2	TITLE-ABS-KEY (ovary AND syndrome, AND polycystic)
#3	TITLE-ABS-KEY (syndrome, AND polycystic AND ovary)
#4	TITLE-ABS-KEY (stein-leventhal AND syndrome)
#5	TITLE-ABS-KEY (stein AND leventhal AND syndrome)
#6	TITLE-ABS-KEY (syndrome, AND stein-leventhal)
#7	TITLE-ABS-KEY (sclerocystic AND ovary AND syndrome)
#8	TITLE-ABS-KEY (ovarian AND degeneration, AND sclerocystic)
#9	TITLE-ABS-KEY (sclerocystic AND ovary AND syndrome)
#10	TITLE-ABS-KEY (polycystic AND ovarian AND syndrome)
#11	TITLE-ABS-KEY (ovarian AND syndrome, AND polycystic)
#12	TITLE-ABS-KEY (polycystic AND ovary AND syndrome 1)
#13	TITLE-ABS-KEY (sclerocystic AND ovaries)
#14	TITLE-ABS-KEY (ovary, AND sclerocystic)
#15	TITLE-ABS-KEY (sclerocystic AND ovary)
#16	TITLE-ABS-KEY (ovary AND polycystic AND disease)
#17	TITLE-ABS-KEY (polycystic AND ovary)
#18	TITLE-ABS-KEY (pco)
#19	TITLE-ABS-KEY (pcos)
#20	#1 OR #2 OR #3 OR #4 OR #5 OR #6 OR #7 OR #8 OR #9 OR #10 OR #11 OR #12 OR #13 OR #14OR #15 OR #16 OR #17OR #18 OR #19
#21	TITLE-ABS-KEY (curcumin)
#22	TITLE-ABS-KEY (nanocurcumin)
#23	TITLE-ABS-KEY (theracurmin)
#24	TITLE-ABS-KEY (cur-cuminoid)
#25	TITLE-ABS-KEY (curcuminoid)
#26	TITLE-ABS-KEY (curcuminoids)
#27	TITLE-ABS-KEY (curqfen)
#28	TITLE-ABS-KEY (turmeric AND yellow)
#29	TITLE-ABS-KEY (yellow, AND turmeric)
#30	TITLE-ABS-KEY (curcumin AND phytosome)
#31	TITLE-ABS-KEY (phytosome, AND curcumin)

#32	TITLE-ABS-KEY (diferuloylmethane)
#33	TITLE-ABS-KEY (mervia)
#34	TITLE-ABS-KEY (curcuma)
#35	TITLE-ABS-KEY (curcumas)
#36	TITLE-ABS-KEY (curcuma AND zedoaria)
#37	TITLE-ABS-KEY (curcuma AND zedoarias)
#38	TITLE-ABS-KEY (curcumalonga)
#39	TITLE-ABS-KEY (zedoaria, AND curcuma)
#40	TITLE-ABS-KEY (zedoary AND zedoaria)
#41	TITLE-ABS-KEY (zedoary AND zedoarias)
#42	TITLE-ABS-KEY (zedoaria, AND zedoary)
#43	TITLE-ABS-KEY (curcuma AND longa)
#44	TITLE-ABS-KEY (curcuma AND longas)
#45	TITLE-ABS-KEY (longa, AND curcuma)
#46	TITLE-ABS-KEY (tumeric)
#47	TITLE-ABS-KEY (tumerics)
#48	TITLE-ABS-KEY (turmeric)
#49	TITLE-ABS-KEY (turmerics)
#50	#21 OR #22 OR #23 OR #24 OR #25 OR #26 OR #27 OR #28 OR #29 OR #30 OR #31 OR #32 OR #33 OR #34 OR #35 OR #36 OR #37 OR #38 OR #39 OR #40 OR #41 OR #42 OR #43 OR #44 OR #45 OR #46 OR #47 OR #48 OR #49
#51	#20 AND #50

Source: Web of Science; Searched on: May 2022; Results: 46

Search	Query
#1	TS="Polycystic Ovary Syndrome"
#2	TS="Ovary Syndrome, Polycystic"
#3	TS="Syndrome, Polycystic Ovary"
#4	TS="Stein-Leventhal Syndrome"
#5	TS="Stein Leventhal Syndrome"
#6	TS="Syndrome, Stein-Leventhal"
#7	TS="Sclerocystic Ovary Syndrome"
#8	TS="Ovarian Degeneration, Sclerocystic"
#9	TS="Sclerocystic Ovary Syndrome"
#10	TS="Polycystic Ovarian Syndrome"
#11	TS="Ovarian Syndrome, Polycystic"
#12	TS="Polycystic Ovary Syndrome 1"
#13	TS="Sclerocystic Ovaries"
#14	TS="Ovary, Sclerocystic"
#15	TS="Sclerocystic Ovary"
#16	TS="ovary polycystic disease"
#17	TS="polycystic ovary"
#18	TS="PCO"
#19	TS="PCOS"

#20	#1 OR #2 OR #3 OR #4 OR #5 OR #6 OR #7 OR #8 OR #9 OR #10 OR #11 OR #12 OR #13 OR #14 OR #15 OR #16 OR #17 OR #18 OR #19
#21	TS="Curcumin"
#22	TS="nanocurcumin"
#23	TS="Theracurmin"
#24	TS="cur-cuminoid"
#25	TS="Curcuminoid"
#26	TS="Curcuminoids"
#27	TS="curqfen"
#28	TS="Turmeric Yellow"
#29	TS="1,6-Heptadiene-3,5-dione, 1,7-bis(4-hydroxy-3-methoxyphenyl)-, (E,E)-"
#30	TS="Yellow, Turmeric"
#31	TS="Curcumin Phytosome"
#32	TS="Phytosome, Curcumin"
#33	TS="Diferuloylmethane"
#34	TS="Mervia"
#35	TS="Curcuma"
#36	TS="Curcumas"
#37	TS="Curcuma zedoaria"
#38	TS="Curcuma zedoarias"
#39	TS="curcumalonga"
#40	TS="zedoaria, Curcuma"
#41	TS="Zedoary zedoaria"
#42	TS="Zedoary zedoarias"
#43	TS="zedoaria, Zedoary"
#44	TS="Curcuma longa"
#45	TS="Curcuma longas"
#46	TS="longa, Curcuma"
#47	TS="Tumeric"
#48	TS="Tumerics"
#49	TS="Turmeric"
#50	TS="Turmerics"
#51	#21 OR #22 OR #23 OR #24 OR #25 OR #26 OR #27 OR #28 OR #29 OR #30 OR #31 OR #32 OR #33 OR #34 OR #35 OR #36 OR #37 OR #38 OR #39 OR #40 OR #41 OR #42 OR #43 OR #44 OR #45 OR #46 OR #47 OR #48 OR #49 OR #50
#52	#20 AND #51

Search in Chinese: CBM; Results: 5

**Search**	**Query**
#1	“duonangluanchaozonghezheng”[Unweighted: extended]
#2	duonangluanchaozonghezheng
#3	duonangluanchaozonghezheng
#4	Stein-Leventhalzonghezheng
#5	Stein-Leventhalzonghezheng

#6	duonangluanchao
#7	PCO
#8	PCOS
#9	#1 OR #2 OR #3 OR #4 OR #5 OR #6 OR #7 OR #8
#10	“jianghuangsu”[Unweighted: extended]
#11	jianghuangsu
#12	jianghuang
#13	#10 OR #11 OR #12
#14	“suijiduizhaoshiyan”[Unweighted: extended]
#15	suijiduizhaoshiyan
#16	suiji
#17	#14 OR #15 OR #16
#18	#9 AND #13 AND #17

Search in Chinese: CNKI; Results: 24

TKA=(duonangluanchaozonghezheng+duonangluanchaozonghezheng+Stein-Leventhalzonghezheng+Stein-Leventhalzonghezheng+duonangluanchao+PCO+PCOS) AND TKA=(jianghuangsu+jianghuang)

Search in Chinese: Wanfang Database; Results: 18

Zhuti:(duonangluanchaozonghezheng+duonangluanchaozonghezheng+Stein-Leventhalzonghezheng+Stein-Leventhalzonghezheng+duonangluanchao+PCO+PCOS) * Zhuti:(jianghuangsu+jianghuang)

Search in Chinese: VIP database; Results: 4

(M=duonangluanchaozonghezheng+duonangluanchaozonghezheng+Stein-Leventhalzonghezheng+Stein-Leventhalzonghezheng+duonangluanchao+PCO+PCOS)*(M=jianghuangsu+jianghuang)

Clinical Trials: 0

Condition or disease: Polycystic Ovary Syndrome OR Ovary Syndrome, Polycystic OR Syndrome, Polycystic Ovary OR Stein-Leventhal Syndrome OR Stein Leventhal Syndrome OR Syndrome, Stein-Leventhal OR Sclerocystic Ovary Syndrome OR Ovarian Degeneration, Sclerocystic OR Sclerocystic Ovary Syndrome OR Polycystic Ovarian Syndrome OR Ovarian Syndrome, Polycystic OR Polycystic Ovary Syndrome 1 OR Sclerocystic Ovaries OR Ovary, Sclerocystic OR Sclerocystic Ovary OR ovary polycystic disease OR polycystic ovary OR PCO OR PCOS

Intervention/treatment: Curcumin OR nanocurcumin OR Theracurmin OR cur-cuminoid OR Curcuminoid OR Curcuminoids OR curqfen OR Turmeric Yellow OR 1,6-Heptadiene-3,5-dione, 1,7-bis(4-hydroxy-3-methoxyphenyl)-, (E,E)- OR Yellow, Turmeric OR Curcumin Phytosome OR Phytosome, Curcumin OR Diferuloylmethane OR Mervia OR Curcuma OR Curcumas OR Curcuma zedoaria OR Curcuma zedoarias OR curcumalonga OR zedoaria, Curcuma OR Zedoary zedoaria OR Zedoary zedoarias OR zedoaria, Zedoary OR Curcuma longa OR Curcuma longas OR longa, Curcuma OR Tumeric OR Tumerics OR Turmeric OR Turmerics

The Chinese Clinical Trials Registry : 0

Subject of registration: duonangluanchaozonghezheng
