# Supplementary material for: Therapeutic effect and safety of curcumin in women with PCOS: A systematic review and meta-analysis
Source: Front Endocrinol (Lausanne). 2022 Oct 27;13:1051111. doi: 10.3389/fendo.2022.1051111 (PMC9646792; doi:10.3389/fendo.2022.1051111)
Supplement: Supplementary file 2 [file DataSheet_2.pdf]

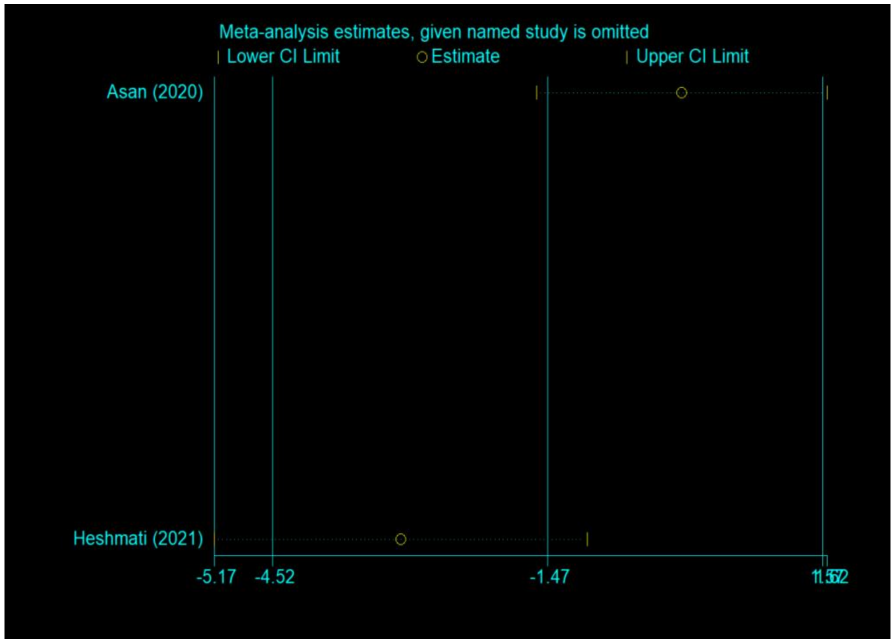
Meta-analysis estimates, given named study is omitted
Lower CI Limit
Estimate
Upper CI Limit
Asan (2020)
Heshmati (2021)
-5.17
-4.52
-1.47

a

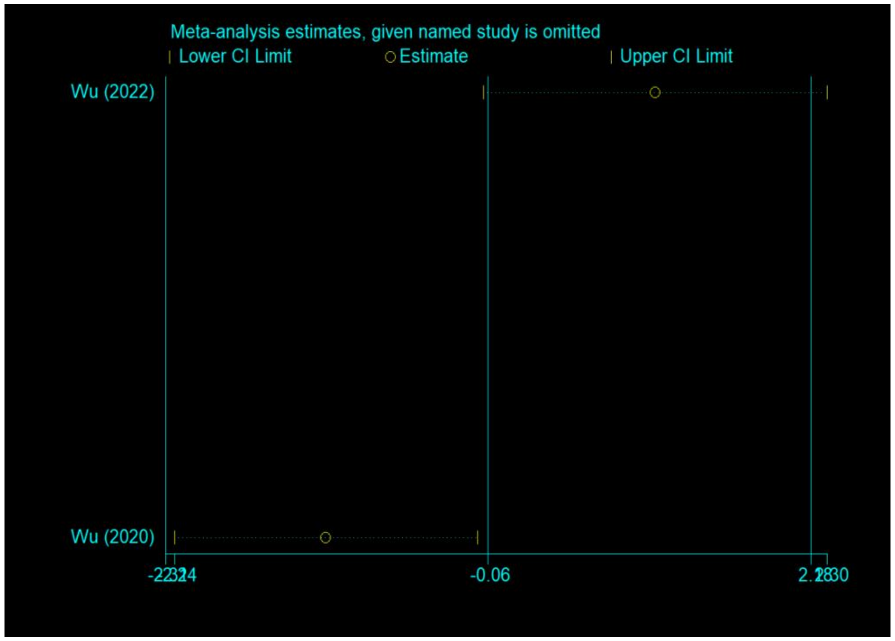
Meta-analysis estimates, given named study is omitted
Lower CI Limit
Estimate
Upper CI Limit
Wu (2022)
Wu (2020)
-0.06

b

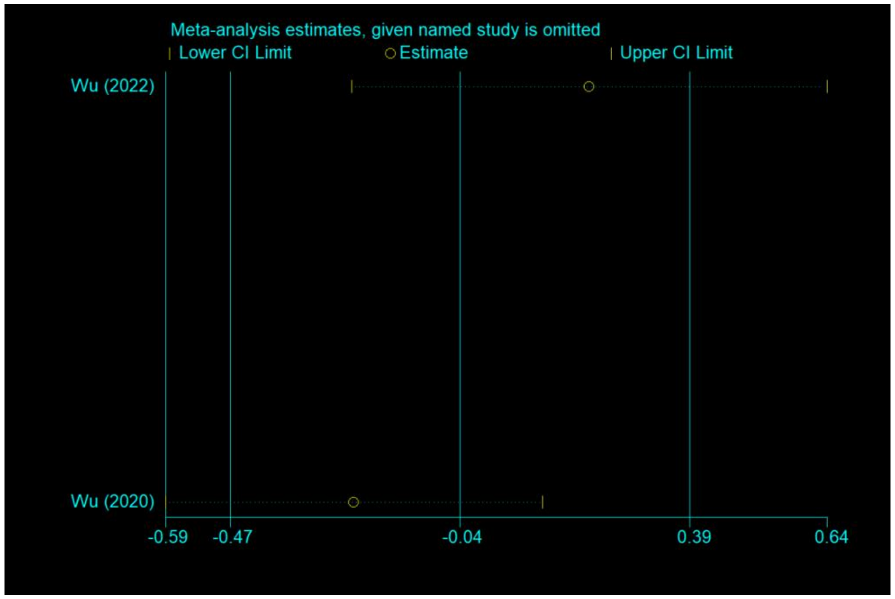
Meta-analysis estimates, given named study is omitted
Lower CI Limit
Estimate
Upper CI Limit
Wu (2022)
Wu (2020)
-0.59
-0.47
-0.04
0.39
0.64

c

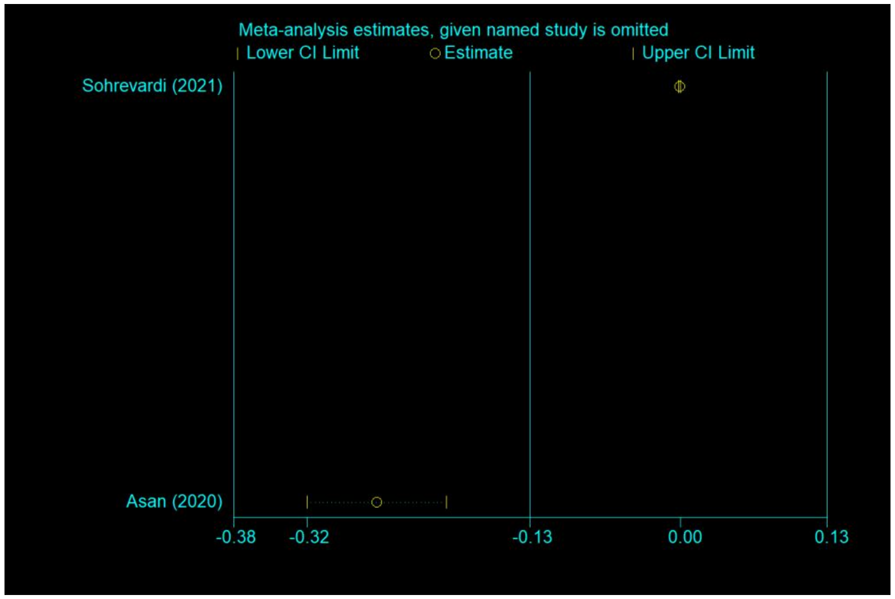
Meta-analysis estimates, given named study is omitted
Lower CI Limit
Estimate
Upper CI Limit
Sohrevardi (2021)
Asan (2020)
-0.38
-0.32
-0.13
0.00
0.13

d

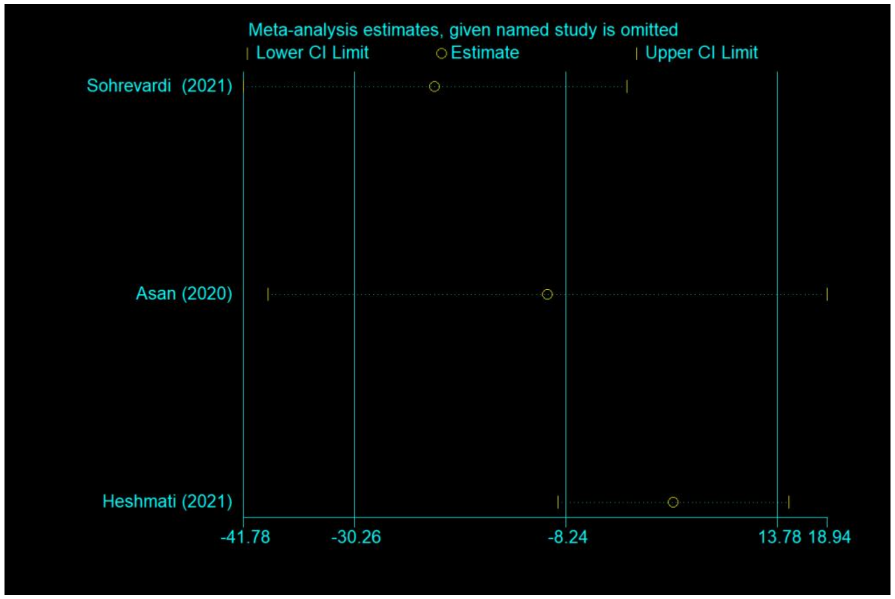

Meta-analysis estimates, given named study is omitted
Lower CI Limit
Estimate
Upper CI Limit
Sohrevardi (2021)
Asan (2020)
Heshmati (2021)
-41.78
-30.26
-8.24
13.78
18.94

e

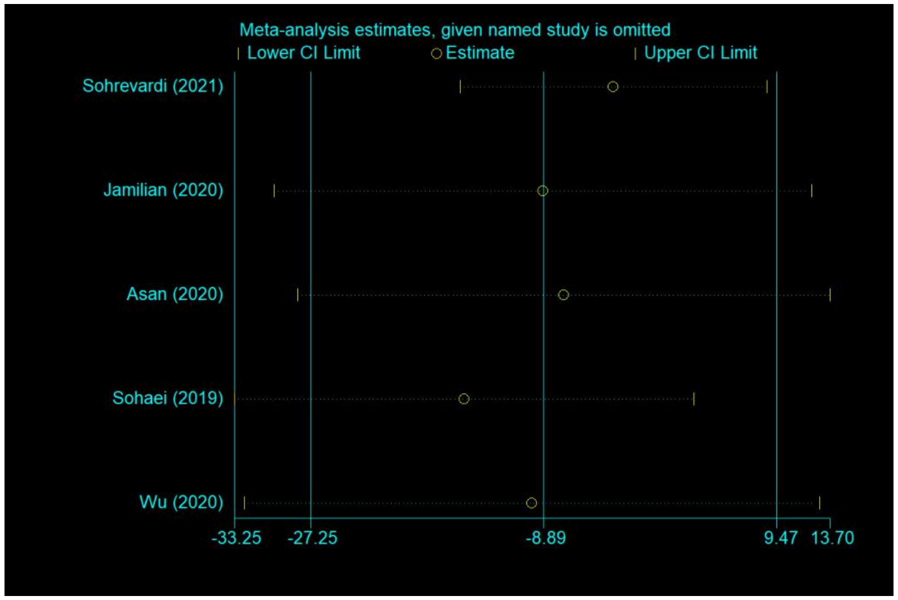

Meta-analysis estimates, given named study is omitted
Lower CI Limit
Estimate
Upper CI Limit
Sohrevardi (2021)
Jamilian (2020)
Asan (2020)
Sohaei (2019)
Wu (2020)
-33.25
-27.25
-8.89
9.47
13.70

f

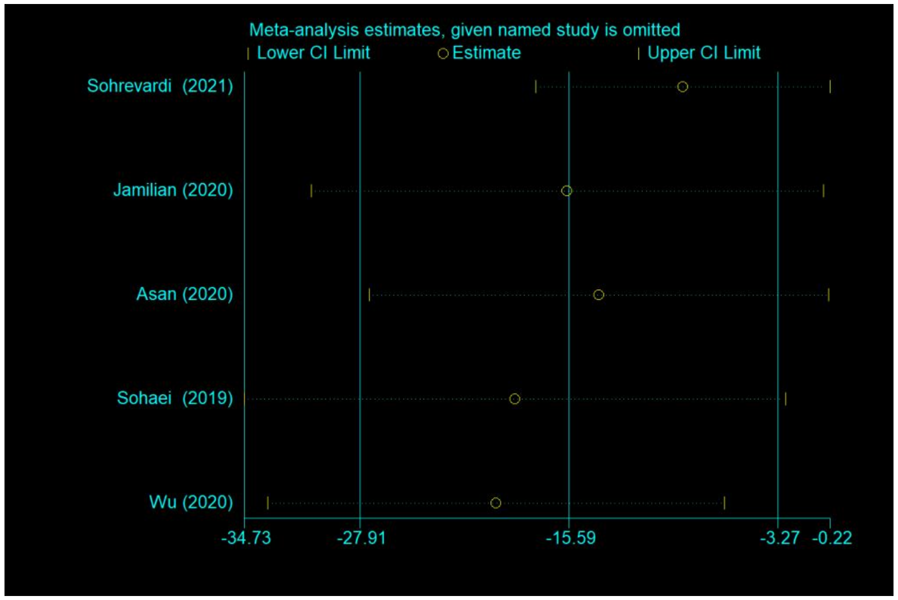

Meta-analysis estimates, given named study is omitted
Lower CI Limit
Estimate
Upper CI Limit
Sohrevardi (2021)
Jamilian (2020)
Asan (2020)
Sohaei (2019)
Wu (2020)
-34.73
-27.91
-15.59
-3.27
-0.22

g

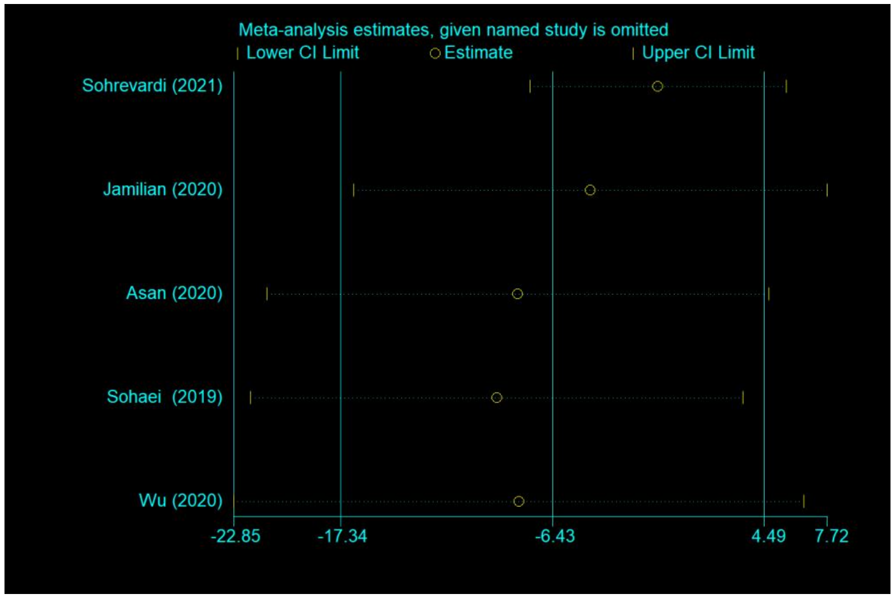

Meta-analysis estimates, given named study is omitted
Lower CI Limit
Estimate
Upper CI Limit
Sohrevardi (2021)
Jamilian (2020)
Asan (2020)
Sohaei (2019)
Wu (2020)
-22.85
-17.34
-6.43
4.49
7.72

h

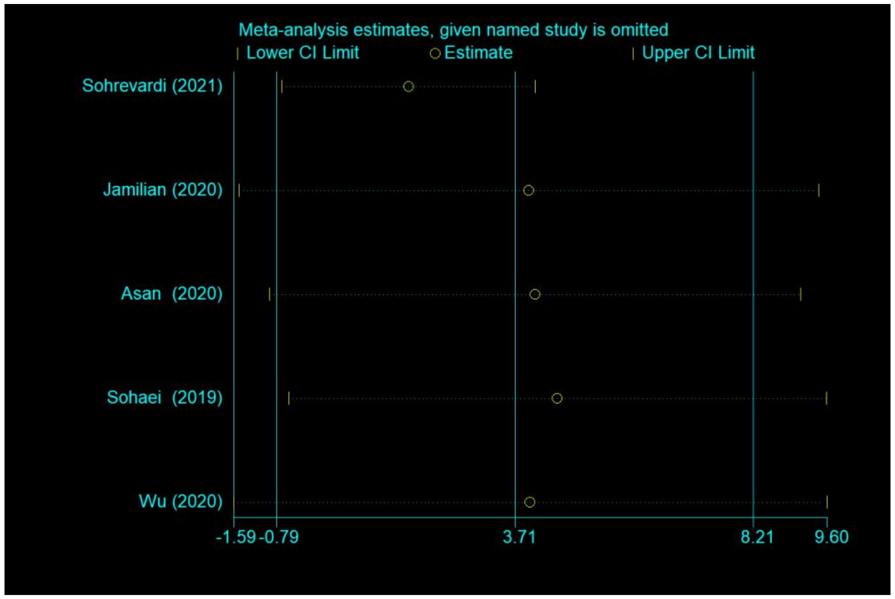

i

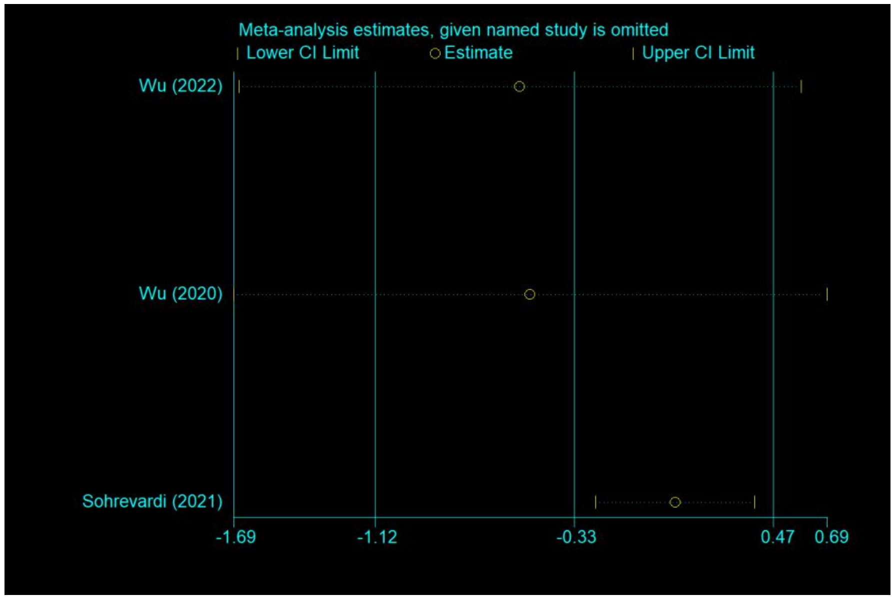

j

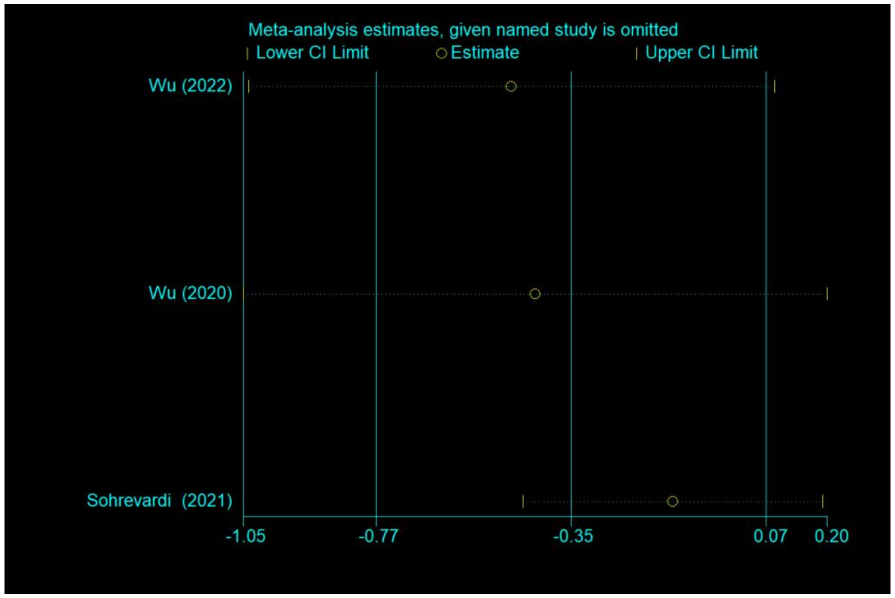

k

Supplementary Appendix 2 Sensitivity analysis curcumin on WC (a), Glu 120 (b), HbA1c (c), T (d), DHEA (e), TG (f), TC (g), LDL-C (h), HDL-C (i), ALT (j) and

AST (k) for PCOS to investigate the effects of each study on pooled standardized mean differences estimates.
