# Supplementary material for: Therapeutic effect and safety of curcumin in women with PCOS: A systematic review and meta-analysis
Source: Front Endocrinol (Lausanne). 2022 Oct 27;13:1051111. doi: 10.3389/fendo.2022.1051111 (PMC9646792; doi:10.3389/fendo.2022.1051111)
Supplement: Supplementary file 3 [file DataSheet_3.pdf]

PRISMA 2020 Checklist

Section and Topic	Item #	Checklist item	Location where item is reported
**TITLE**			
Title	1	This report is identified as a systematic review.	Page 1
**ABSTRACT**			
Abstract	2	The abstract includes background; information sources; the methods used to present and synthesise results; total number of included studies; results for main outcomes and the summary estimate and credible interval; general interpretation of the results and important implications; registration number.	Page 2-3
**INTRODUCTION**			
Rationale	3	Described in the introduction.	Page 3-4
Objectives	4	Stated in the introduction.	Page 3-4
**METHODS**			
Eligibility criteria	5	In this paper, we clearly put forward the inclusion and exclusion criteria.	Page 5
Information sources	6	We searched Chinese database (Chinese National Knowledge Infrastructure, China Biology Medicine Databases, VIP database, and so on), English database (PubMed, Web of Science, Embase, and so forth) and additional search of grey literature and missing literature to screen out eligible literatures published up to May 2022.	Page 4
Search strategy	7	We have submitted search strategies for all databases mentioned in this article in Supplementary Appendix 1.	Supplementary Appendix 1
Selection process	8	Two investigators independently screened each record and each report retrieved on the basis of inclusion, and any disagreement was resolved by discussion.	Page 5
Data collection process	9	Two researchers independently collected data from each report and cross-checked the results to ensure the data accuracy. Any discrepancy was resolved through discussion to reach consensus.	Page 5
Data items	10a	We collected every outcome parameter and adverse effect from each study.	Page 5
	10b	The following parameters were collected from each study: basic information of the articles, participants, curcumin characteristics and comparison methods. For studies with missing or ambiguous data, if possible, we will attempt to contact the first or corresponding author via telephone or email for clarification or addition to ensure the integrity of the data.	Page 5
Study risk of bias assessment	11	Two authors used the Cochrane risk of bias tool to assess methodological quality of RCTs. Each reviewer appraised bias according to the specific content within each item, designating a low, high, or unclear risk of bias by answering yes, no or unclear. Disagreements between the two reviewers were resolved through discussion or by consulting a third author.	Page 5
Effect measures	12	For dichotomous variables, the odds ratio (OR) with corresponding 95% confidence intervals (CIs) was calculated to summarize the difference between the groups. For continuous data, the results were presented as weighted mean difference (WMD) together with 95% CI of changes before and after the therapy in the curcumin group with those in the control group. Since some studies used different measures for the same outcome (eg, AST and ALT), we calculated the standardized mean difference (SMD).	Page 5-6
Synthesis methods	13a	Not mentioned.	
	13b	Not mentioned.	
	13c	Not mentioned.	
	13d	We used Stata Software, version 14.0 (StataCorp) for systematic reviews of interventions. Heterogeneity among the included studies was estimated using Q statistic and the I^2 statistic, results were deemed as low heterogeneity ($I^2 < 25\%$), medium heterogeneity ($I^2 = 25\%$-50%), or high heterogeneity ($I^2 > 50\%$). Owing to the clinical heterogeneity inherent in our data such as ethnic differences, different use of curcumin preparations as well as duration of treatment, and so forth, random-effects models were performed for calculating pooled effect measures.	Page 5-6

PRISMA 2020 Checklist

Section and Topic	Item #	Checklist item	Location where item is reported
	13e	Not mentioned.	
	13f	We have submitted sensitivity analysis in Supplementary Appendix 2.	Supplementary Appendix 2
Reporting bias assessment	14	We assessed methodological quality of RCTs, which included the following seven specified domains: random sequence generation, allocation concealment, blinding of participants and personnel, blinding of outcome assessment, incomplete outcome data, selective reporting, and other bias.	Page 5
Certainty assessment	15	Not mentioned.	
**RESULTS**			
Study selection	16a	We described the results of the search and selection process, from the number of records identified in the search to the number of studies included in the review. The literature selection process is depicted in Figure 1.	Figure 1
	16b	We have explained this item in detail in Figure 1.	Figure 1
Study characteristics	17	The main characteristics of the included studies in the present meta-analysis are described in Table 1.	Table 1
Risk of bias in studies	18	Figure 2 summarizes the risk of bias for each included study according to the pre-defined criteria in Cochrane handbook.	Figure 2
Results of individual studies	19	We used forest plots to present summary statistics for each group and effect estimates and its precision (Figure 3-8).	Figure 3-8
Results of syntheses	20a	We briefly summarized the characteristics and risk of bias for each synthesis.	Page 7-9
	20b	We listed the results of all statistical syntheses, as well as each summary estimate and its precision and measures of statistical heterogeneity.	Page 7-9
	20c	Not mentioned.	
	20d	Based on the results of our meta-analysis, we performed a sensitivity analysis for outcomes with high heterogeneity: WC, Glu 120, HbA1c, T, DHEA, TG, TC, LDL-C, HDL-C, ALT and AST. The results of sensitivity analyses showed that all the points fall in the confidence interval, indicating that none of the individual studies affect the final conclusion obviously (Supplementary Appendix 2).	Supplementary Appendix 2
Reporting biases	21	We assessed the risk of bias due to missing results (arising from reporting biases) for each synthesis assessed.	Page 9
Certainty of evidence	22	We assessed the certainty (or confidence) in the body of evidence for each outcome assessed.	Page 7-9
**DISCUSSION**			
Discussion	23a	In the context of other evidence, we provide a general interpretation of the therapeutic efficacy and safety results of curcumin in patients with PCOS.	Page 9
	23b	We discussed four limitations included in the review.	Page 11-12
	23c	Not mentioned.	
	23d	The results of this meta-analysis are inspiring and provide evidence supporting the potential effectiveness and safety of curcumin in orchestrating the inflammatory microenvironment and reducing the risk of abnormalities of glucose and lipid metabolism and obesity in patients with PCOS. However, the strength of this conclusion is tempered by the dearth of large-scale, high-quality reference datasets and the significant number of studies on this topic. Indeed, the effect sizes reported in this analysis merit further evaluation in a larger, well-designed, high-quality prospective randomized clinical trial.	Page12
**OTHER INFORMATION**			

PRISMA 2020 Checklist

Section and Topic	Item #	Checklist item	Location where item is reported
Registration and protocol	24a	PROSPERO https://www.crd.york.ac.uk/prospero/display_record.php?ID=CRD42022332394, identifier CRD42022332394.	Page 3
	24b	The protocol was not prepared.	
	24c	Not mentioned.	
Support	25	This work is supported by the Young Scientists Project of the National Natural Science Foundation of China (81803945), National Natural Science Foundation of China (82074259), Scientific Research Project of Traditional Chinese Medicine in Heilongjiang Province (ZHY19024), and the Project of Young Innovative Talents in Colleges and Universities in Heilongjiang Province (UNPYSCT-2016216). WS conceptualized the research question. YZ participated in the drawing of tables and figures.	Page 13
Competing interests	26	The research was conducted in the absence of any commercial or financial relationship that could be construed as a potential conflict of interest.	
Availability of data, code and other materials	27	The original contributions presented in the study are included in the article/Supplementary Material. Further inquiries can be directed to the corresponding authors.	Page 12

From: Page MJ, McKenzie JE, Bossuyt PM, Boutron I, Hoffmann TC, Mulrow CD, et al. The PRISMA 2020 statement: an updated guideline for reporting systematic reviews. BMJ 2021;372:n71. doi: 10.1136/bmj.n71

For more information, visit: http://www.prisma-statement.org/
